# Natural Products and Neuroregeneration: Rethinking Discovery Beyond Bioavailability Through Pseudo-Natural Product Design

**DOI:** 10.3390/biomedicines14092009

**Published:** 2026-09-07

**Authors:** Solomon Habtemariam

**Affiliations:** Pharmacognosy Research & Herbal Analysis Services UK, 124 City Road, London EC1V 2NX, UK; s.habtemariam@herbalanalysis.co.uk

**Keywords:** pseudo-natural products, neuroregeneration, axonal regeneration, adult neurogenesis, neuroplasticity, cell painting assay, neurotrophins, Trk signalling

## Abstract

Natural product (NP)-based neuroregeneration research has generated extensive preclinical evidence over the past five decades. Pharmacological activity across core processes of central nervous system (CNS) repair including neurogenesis, axonal regeneration, and neuroplasticity have been documented. Despite consistent observations of neurite outgrowth, neuroprotection, and partial functional recovery in cellular and animal models, translation into durable clinical therapies has remained limited. Neurotrophins such as nerve growth factor (NGF) and brain-derived neurotrophic factor (BDNF) similarly exhibit strong regenerative effects in experimental systems, but even direct central administration has failed to produce sustained long-distance axonal regeneration or stable circuit reconstruction. This suggests that delivery constraints alone do not explain the failure to achieve clinically-relevant functional repair. It is proposed herein that this limitation reflects intrinsic constraints in how regenerative signalling is organised across multiple biological scales. Integrating evidence from in vitro and in vivo injury models, we can introduce a Target–Mechanism–Network (T-M-N) approach that systematically maps NPs activity onto a hierarchical regulatory architecture. Across diverse NPs classes, ~55 recurrent molecular targets cluster into 10 functional mechanisms, which converge into four higher-order network control regimes governing energetic competence, regenerative signalling capacity, redox-immune balance, and structural plasticity. This analysis reveals that NPs converge on shared regenerative networks but rarely coordinate all required domains within a unified pharmacological programme. They can thus be seen to represent a pre-organised source of evolutionarily selected pharmacophores encoding discrete elements of neuroregenerative network control. On this basis, pseudo-natural product (PNP) design enabled by computational chemistry and phenotypic screening may provide a strategy to recombine these fragments into engineered scaffolds with improved functional selectivity and regenerative coherence. The need to shift drug discovery from optimisation of individual NPs toward architecture-driven design of multi-functional molecules that address the integrated demands of neuroregeneration is discussed.

## 1. The Triple Architecture of Neuroregeneration Challenges

In this manuscript, neuroregeneration is used as an operational term encompassing two principal regenerative processes: axonal recovery (regeneration) and adult neurogenesis. These processes are considered distinct but complementary routes to restoration of neuronal structure and function. Neuroprotection is treated separately and is not classified as neuroregeneration in the absence of evidence for a regenerative process. The challenges associated with neuroregeneration are considered across three interconnected levels: the intrinsic capacity of neurons to initiate and sustain regeneration, the extrinsic tissue environment that can restrict or support regenerative responses, and the coordination of these processes across the temporal and spatial dimensions of the injured nervous system.

### 1.1. The Chronological Assembly Line and the Reality of Neuronal Damage: Axonal Recovery

**Figure 1:**
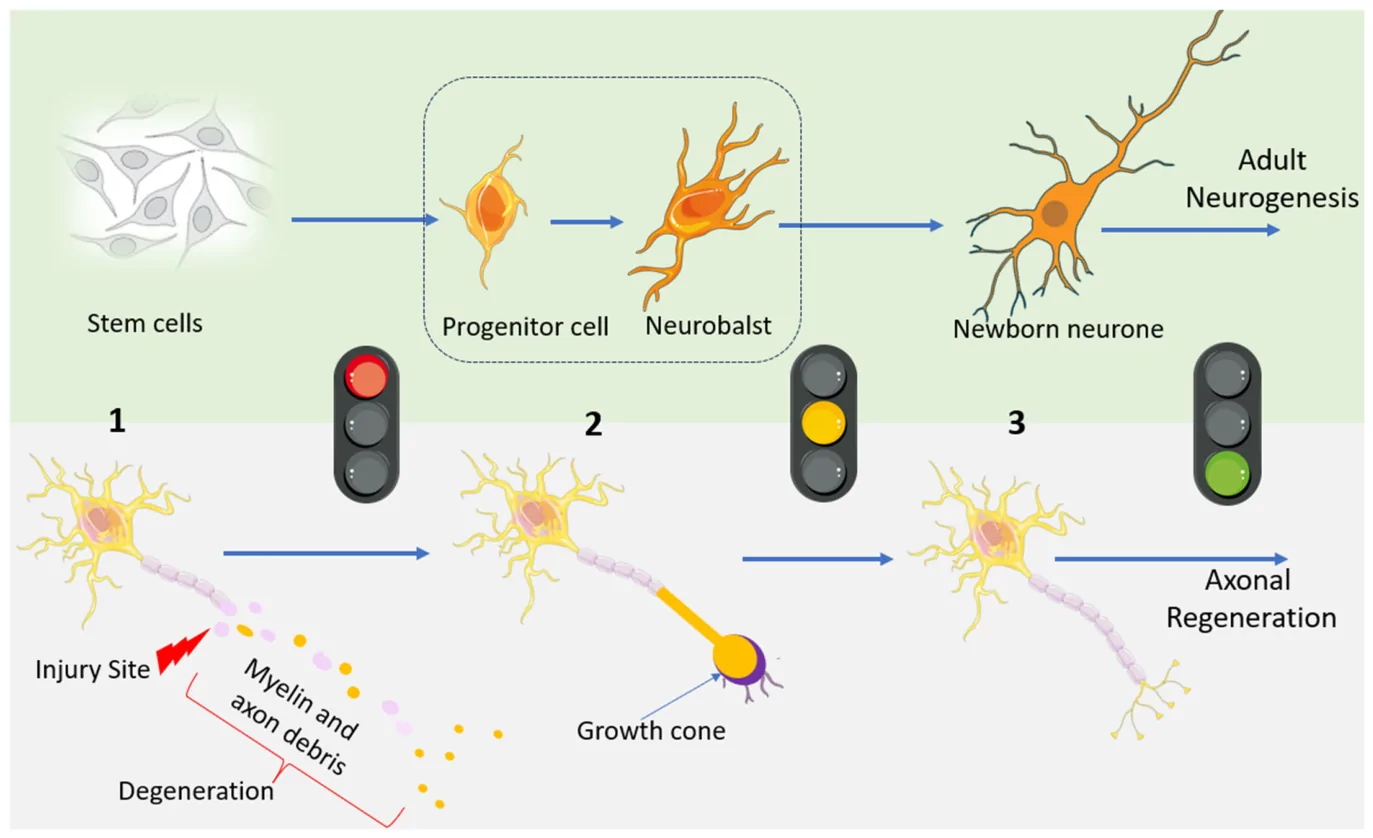
Traffic-light regulation of adult neuroregeneration: Bottlenecks in neurogenesis and axonal repair. The adult CNS severely downregulates regenerative capacity through a traffic-light checkpoint system that restricts the survival of limited neural stem cell pools and damaged fibres. The top panel outlines the three-stage neurogenic lineage from a scarce population of neural stem cells, through neuroblast progenitors, to newborn neurons. The Stage 1-to-2 transition acts as a restrictive red light where environmental factors heavily suppress stem cell activation. Surviving progenitors face a yellow/amber-light checkpoint during the Stage 2-to-3 transition, encountering harsh physical and biochemical selection barriers that further downregulate cell survival. The final green-light milestone marks mature integration, governed by a strict, activity-dependent use it or lose it wiring principle. The lower panel depicts the parallel three-stage process of axonal repair from a broken axon with myelin/neuron debris, through growth cone extension, to a fully myelinated neuron. The initial injury site presents a red-light roadblock were debris halts growth cone initiation. Elongation proceeds through a yellow/amber-light checkpoint, slowed by physical scars and biochemical steering barriers. Finally, the green-light phase signifies successful remyelination and functional target wiring, requiring sustained neural activity to prevent axonal retraction.

Functional in vivo neuroregeneration within the injured, ischemic, or degenerating adult mammalian central nervous system (CNS) is an exceptionally complex biological process that operates as a strict and stage-gated chronological assembly line. In the past, classical neurobiology and ethnopharmacology reviews have frequently engaged by isolating neural repair into the narrow domain of embryonic or adult neurogenesis by focusing on cellular proliferation and differentiation within specialised stem cell niches. Clinically relevant recovery should, however, directly confront the immediate catastrophic neuronal damage caused by mechanical impacts. Following physical trauma such as that seen in traumatic brain injury (TBI), existing functional axonal tracts are severed to an extent that the internal microtubule skeleton is disrupted and the damaged growth cone collapses into a non-functional static retraction bulb. Lacking physical structural continuity, distal segments undergo rapid Wallerian degeneration leading to effective severing of the long-distance bioelectric pipelines of the brain [1]. Therefore, before even generating newborn neuroblasts, an effective neuroregeneration system must try to orchestrate the physical repair, growth cone re-assembly, and long-distance elongation of damaged axons through a hostile, disrupted tissue matrix. Insights from recent advances in neuroregeneration science indicate that this intricate architecture is organised into three fundamental parallel pillars that must be satisfied simultaneously to prevent permanent functional deficit. We may refer to these pillars as means for overcoming the Triple Regenerative Barriers (Figure 1).

The first pillar is dedicated to promoting an intrinsic growth machinery which is downregulated in adult neurons. Intrinsic growth promotion requires the deep activation of intracellular gene networks responsible for re-assembling the collapsed growth cone to accelerate structural protein synthesis, and driving rapid tubulin polymerisation to extend the physical axon shaft [2,3,4]. The growth process functions as an intrinsic engine of the triple regenerative niche, triggering a profound metabolic and genetic shift that transitions the injured neuron into an embryonic-like state of active elongation (Figure 1). This phase is biochemically orchestrated by the deep activation of the PI3K/Akt/mTORC1 (phosphoinositide 3-kinase/protein kinase/mammalian target of rapamycin complex 1) axis, which silences endogenous breaks like PTEN (phosphatase and tensin homolog) to accelerate macro-molecular biosynthesis, initiate localised translation at the lesion site, and fuel the production of membrane lipids and structural proteins [5,6]. Simultaneously, retrograde injury signalling via the DLK/JNK (dual leucine-zipper kinase/c-Jun N-terminal kinase) cascade and elevated intracellular cAMP (cyclic adenosine monophosphate) pools activate the JAK/STAT3 (Janus kinase/signal transducer and activator of transcription 3) and PKA/CREB (protein kinase A/cAMP response element-binding protein) pathways, orchestrating an epigenetic rewriting of the nucleus that upregulates vital regeneration-associated genes (RAGs) like growth-associated protein-43 (GAP-43) [7,8]. At the leading edge, these convergent transcriptional and translational programmes physically re-assemble the collapsed growth cone by inactivating GSK-3β (glycogen synthase kinase-3β), which unleashes microtubule-stabilising proteins like CRMP2 (collapsin response mediator protein-2) to stimulate rapid tubulin polymerisation, while a shift toward GTPases Rac1 and Cdc42 signalling enhances actin treadmilling to physically extend the new axon shaft [9,10].

The second pillar is dedicated to bypass the key inhibitory barriers for neuroregeneration. This is what we call a stage of active neutralisation and bypassing of external inhibitory barriers within the scarred extracellular matrix. The barrier bypass functions as the essential mechanism for overcoming Triple Regenerative Barriers, strategically neutralising the chemical and physical walls erected by the scarred extracellular matrix. At the lesion site, the damaged growth cone is confronted by an aggressive barrier of oligodendrocyte-derived myelin-associated inhibitors (Nogo-A, MAG, and OMgp) and scar-embedded chondroitin sulphate proteoglycans (CSPGs) [11]. These inhibitory ligands bind directly to specialised axonal receptor complexes, principally the Nogo receptor-1 (NgR1)/p75NTR/LINGO-1 triad and the tyrosine phosphatase (PTP) such as PTPσ and leukocyte common antigen-related (LAR) [12]. The receptors are mainly expressed in oligodendrocytes as well as in neurons and microglia. This ligation triggers a catastrophic signalling convergence that overstimulates the small GTPase RhoA and its downstream effector ROCK (Rho-associated kinase), while simultaneously suppressing Rac1 and Cdc42. Studies also suggest that MAG and OMgp synergise with Nogo-A to restrict axonal growth and neurological recovery after spinal cord trauma in experimental models [13]. Intracellularly, ROCK directly phosphorylates LIM kinase, which inactivates the actin-severing protein cofilin, while also activating myosin II motors to induce massive actomyosin contraction [14]. Deprived of local translation and structurally paralysed by this internal actin collapse and microtubule destabilisation, the axon remains permanently locked in its severed state unless these receptor-mediated inhibitory breaks are bypassed. As discussed in Section 4, axonal regeneration following traumatic injury also encounters significant physical and biochemical barriers. These include disruption of the blood–brain barrier (BBB), alterations to the extracellular matrix (ECM), and the consequences of excessive inflammatory and oxidative stress responses, all of which create a hostile environment for axonal regrowth and functional recovery.

The third pillar of axonal recovery is dedicated to the plasticity and wiring architecture which dictates the downstream functional fate of both newly extended axons and surviving collateral tracts. It represents the definitive functional phase of overcoming the Triple Regenerative Barriers to determine the long-term survival and network integration of both newly extended axons and sprouting collateral tracts. Once an axon successfully bypasses extracellular barriers, its downstream fate depends on precise chemotropic guidance, activity-dependent synaptogenesis, and the establishment of functional long-term potentiation [15,16]. This critical wiring phase is biochemically orchestrated by neurotrophic signalling, principally the binding of the brain-derived neurotrophic factor (BDNF) to tropomyosin-related kinase receptor type B (TrkB) receptors, which recruits the PLCγ/PKC (phospholipase C gamma/protein kinase C) and PI3K/Akt pathways to promote growth cone steering, neurotransmitter receptor clustering, and local vesicle recruitment [17]. In the glutamatergic synapses, for example, activation of postsynaptic NMDA (*N*-methyl-*D*-aspartate) receptors initiates a localised calcium influx that activates CaMKII (calcium/calmodulin-dependent protein kinase II) to trigger retrograde neurotrophin signalling, and induction of structural remodelling of dendritic spines [18]. Without this sustained bioelectric activity and neurotrophic support, the newly formed circuit fails to stabilise. In fact, the lack of synaptic feedback can trigger pro-apoptotic pruning cascades including the DLK (Dual Leucine-zipper Kinase)/bax/caspase-3 axis and complement-mediated microglial phagocytosis which targets the unwired projection for permanent removal [19].

### 1.2. The Chronological Assembly Line and the Reality of Cellular Loss: Adult Neurogenesis

While repairing damaged axonal pipelines handles structural continuity, complete functional restoration following acute ischemia, trauma, or progressive degeneration requires the replacement of entirely lost neuronal cell bodies. To mirror the axonal track, adult neurogenesis operates along the exact same stage-gated chronological assembly line within the Triple Regenerative Barriers (Figure 1), but with shifting focus from elongation to cellular birth, migration, and functional integration. When mechanical or pathological injury destroys parenchymal networks, generating a functional neuron requires neural stem and progenitor cells to break their deep quiescence, cross hostile inflammatory borders, and physically rewire themselves into surviving circuits. This parallel cellular architecture is governed by its own strict, three core pillars of the signalling network to overcome physical and biochemical barriers:

The first pillar of overcoming the Triple Regenerative Barriers in adult neurogenesis is the neurogenic growth process that functions as the proliferative engine of the niche to induce quiescent adult neural progenitor cells to enter the cell cycle within specialised microenvironments such as the subventricular zone or subgranular zone. The first hurdle is, unlike in earlier or embryonic development, the limited proliferative and differentiation capacity of neural stem and progenitor cells in the adult CNS. This initial phase is biochemically triggered by the downregulation of endogenous Notch signalling, which breaks stem cell dormancy, and the concurrent overactivation of the Wnt/β-catenin (wingless-related integration site (Wnt)/β-catenin) and sonic hedgehog (Shh) cascades [20,21,22]. These convergent pathways drive the asymmetric division of neural progenitor cells into highly proliferative intermediate progenitors and transit-amplifying cells. Simultaneously, transcriptional reprogramming occurs through the upregulation of proneural basic helix-loop-helix (bHLH) factors, including Ascl1 and NeuroD1, which epigenetically lock the newly generated cells into a committed neuronal lineage [23,24], promoting macromolecular biosynthesis and setting the stage for structural morphological development.

The second pillar of overcoming the Triple Regenerative Barriers of neurogenesis is dedicated to neuroblast barrier bypass as described in axonal regeneration. Once established, the fragile and nascent neuroblasts must execute a long-distance tangential and radial migration out of their protected niches to reach the remote and highly hostile site of parenchymal tissue damage. This cellular migration is directly blocked by the identical chemical and physical barriers that paralyse growth cones, namely dense reactive astrocytic walls, rigid CSPG meshes, and myelin-associated inhibitors. To bypass this inhibitory blockade and cross the scar tissue matrix, migrating neuroblasts downregulate internal RhoA/ROCK signalling to maintain forward membrane fluidity, while upregulating the expression and focal secretion of matrix metalloproteinases (MMPs) [25], specifically MMP-2 and MMP-9, to physically digest ECM restrictions. Navigating through this cleared path is driven by a localised chemotropic gradient, where the chemokine SDF-1 (CXCL12) secreted by the injured tissue binds directly to CXCR4 receptors on the migrating neuroblast [26], pulling the cells forward through the scarred landscape. Similarly, as discussed in Section 4, adult neurogenesis following traumatic injury or neurodegenerative diseases (NDs) is constrained by both intrinsic and extrinsic factors. These include disruption of the neurogenic niche due to BBB breakdown and ECM remodelling, and the detrimental effects of persistent inflammation and oxidative stress, which all impair the generation, survival, and integration of new neurons.

The third pillar of overcoming adult neurogenesis barriers involve functional wiring and integration as described for axonal repair. This dictates the post-migratory functional fate and long-term survival of the newly arrived neuroblasts within the host neural network. To escape programmed cell death and secure circuit permanence, the newborn neuron must successfully execute an electrophysiological and structural maturation process. This wiring phase is biochemically initiated by a critical developmental bioelectric shift, operating alongside sustained BDNF/TrkB signalling to stimulate extensive dendritic arborisation and spinogenesis. If the newly integrated neuron fails to receive sufficient activity-dependent synaptic feedback and achieve functional long-term potentiation, the absence of bioelectric purpose triggers pro-apoptotic cascades—principally the Bax/caspase-3 axis [27]. Hence, unwired cells are targeted for removal by complement-mediated microglial phagocytosis, among others.

### 1.3. Endogenous Polypharmacology in Axon Guidance and Neurogenesis

**Figure 2:**
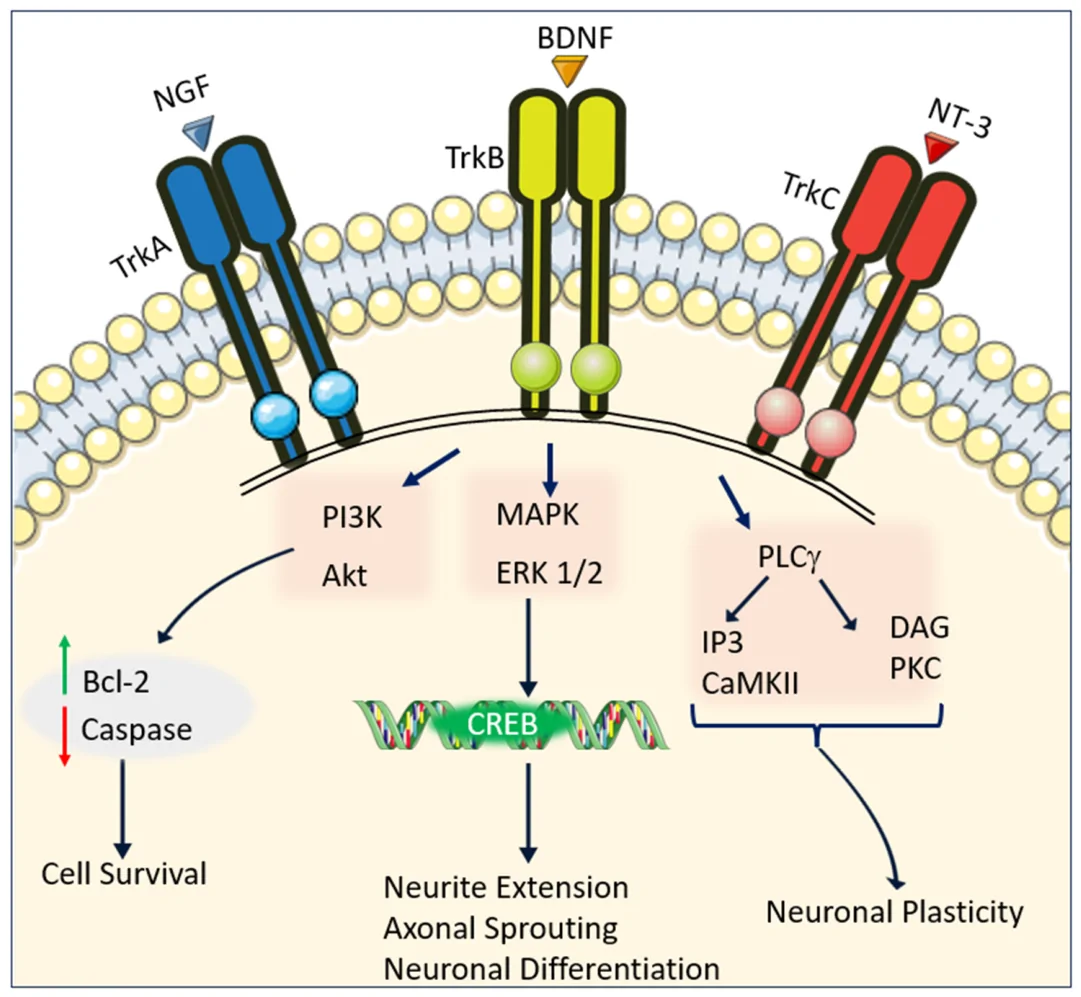
Pleiotropic neuroregenerative actions of neurotrophins through Trk receptor signalling. Neurotrophins, including nerve growth factor (NGF), brain-derived neurotrophic factor (BDNF), and neurotrophin-3 (NT-3), mediate diverse regenerative responses through activation of their cognate tropomyosin receptor kinase (Trk) receptors, TrkA, TrkB, and TrkC, respectively. Neurotrophin binding induces receptor dimerisation and autophosphorylation, leading to activation of three major intracellular signalling cascades. The PI3K/Akt pathway promotes neuronal survival through the inhibition of apoptotic signalling and enhancement of cell viability. The Ras/MAPK/ERK pathway regulates neuronal differentiation, neurite extension, axonal growth, and regenerative remodelling. Activation of PLCγ generates IP_3_ and DAG, resulting in intracellular Ca^2+^ mobilisation and subsequent activation of Ca^2+^/calmodulin-dependent protein kinase II (CaMKII) and protein kinase C (PKC). The signalling pathways contribute to synaptic plasticity, activity-dependent gene expression, neurotransmitter release, and circuit remodelling. These interconnected pathways illustrate the pleiotropic nature of neurotrophin signalling, whereby NGF, BDNF, and NT-3 co-ordinate neuronal survival, axonal regeneration, and synaptic reorganisation to support functional recovery following neural injury.

The mammalian brain’s native solution to the above-mentioned triple developmental challenge relies on the execution of endogenous polypharmacology. Canonical neurotrophins, such as nerve growth factor (NGF) and BDNF, do not operate via clean, single-target molecular mechanisms. They act as master network coordinators designed to simultaneously guide advancing growth cones and govern the fate of newly generated neuroblasts through damaged microenvironments [28]. When a mature, homodimeric BDNF protein docks onto its high-affinity membrane receptor, TrkB, it induces receptor autophosphorylation to trigger three downstream signalling pathways simultaneously (Figure 2).

- The MAPK/ERK (mitogen-activated protein kinase/extracellular signal-regulated kinase) pathway serves as a driving force for primary growth [29,30]. The activation of this pathway promotes the expression of structural transcription factors required for physical axon lengthening and growth cone motility in injured tracts. This exact same cascade also upregulates the bHLH transcription factors, such as NeuroD1 and Ascl1, that dictate migrating neural progenitors to exit the cell cycle and commit to a mature neuronal lineage.
- The PI3K/Akt pathway serves as a driving force for metabolic fuel. It upregulates glucose transporters and activates metabolic switches like PGC-1α (peroxisome proliferator-activated receptor γ coactivator 1-α) to expand mitochondrial biomass [31]. This metabolic surge supplies the high-energy ATP required to drive actin filament assembly at the advancing axonal tip, while also providing the immense biosynthetic energy needed to power the intense, migratory cytoskeleton reshaping of newborn neuroblasts traversing the parenchymal injury site.
- The PLCγ/PKC axis promotes the barrier bypass and plasticity intersect. This cascade initiates a rapid intracellular surge of cAMP and PKC to modify internal receptor structures leading to desensitisation of both the advancing growth cone and the migrating neuroblast to extracellular inhibitors like Nogo-A, MAG, and CSPGs. By turning off the internal RhoA/ROCK (the collapse and stop pathways), this endogenous cascade allows the physical axon and/or the nascent neuroblast to ignore myelin and scar brakes, penetrate the glial matrix, and ultimately enhance both synaptic vesicle docking and postsynaptic receptor clustering to promote activity-dependent network plasticity and permanent circuit integration [32,33].

Among the neurotrophin family, NGF, BDNF, and NT-3 (neurotrophin-3) are the most extensively studied and biologically relevant mediators of neurogenesis, neuronal survival, and axonal repair. These neurotrophins exemplify the capacity of endogenous growth factors to orchestrate multiple stages of neural development and regeneration through interactions with their cognate Trk receptors and downstream signalling networks. Although they share common intracellular effectors, including the PI3K/Akt, MAPK/ERK, and PLCγ pathways (Figure 2), each neurotrophin exhibits distinct patterns of expression, receptor selectivity, and functional specialisation within the CNS and PNS. The NGF primarily supports sensory and sympathetic neuronal populations, BDNF is a key regulator of synaptic plasticity and activity-dependent repair in the central nervous system, whereas NT-3 contributes to neuronal differentiation, axonal guidance, and the maturation of diverse neuronal lineages [34]. Furthermore, the expression of these neurotrophins is tightly regulated in both space and time during development and following injury, generating localised signalling environments that influence survival, regeneration, and functional recovery. With the above-mentioned functional and signalling variations, the pleiotropic effects of neurotrophins arise from the integration of many shared signalling pathways with pathology-dependent patterns of ligand availability and receptor expression, a principle that has informed the development of neurotrophin-inspired therapeutic strategies (Figure 2).

### 1.4. The Natural Product Mirror in Dual-Track Neuroregeneration

From a therapeutic perspective, neurotrophins face several fundamental limitations as regenerative drugs. First, they are large protein molecules with negligible oral bioavailability, poor BBB permeability, and susceptibility to rapid proteolytic degradation, necessitating invasive delivery approaches to achieve therapeutically meaningful concentrations within the CNS [35]. Even after successful administration, neurotrophins exhibit limited tissue diffusion and highly localised distribution within the neural parenchyma which restricts their ability to uniformly access complex lesion environments [36]. Furthermore, their biological activity remains entirely dependent upon the availability and integrity of cognate cell-surface receptors which are subject to downregulation under different physiological and pathological conditions [37]. Following trauma, ischemia, or neurodegeneration, receptor expression, trafficking, and signalling competence may become profoundly altered, reducing tissue responsiveness at the time when trophic support is most needed. Consequently, the clinical challenge of neurotrophin therapy extends beyond drug delivery alone and encompasses a broader problem of tissue accessibility, receptor availability, and spatiotemporal signal control within the injured central nervous system.

Chemical compounds encompassing natural products (NPs) as secondary metabolites isolated from plant, fungal, marine, and microbial origins have evolved complex chemical scaffolds that mirror the multi-pronged network approach of neuroregeneration, often in a manner that is conceptually superior to endogenous proteins. Unlike neurotrophins which are large and dependent on the surface density and structural integrity of membrane-bound receptors, a single multi-targeted natural small molecule can entirely bypass membrane receptors. It may diffuse directly through cellular membranes and the BBB to interact with internal signalling hubs. Moreover, as discussed in the following sections, many NPs such as polyphenols, alkaloids, and terpenoids can simultaneously activate internal survival transcription factors, chemically block the upstream RhoA/ROCK cascade to prevent growth cone retraction in axonal regeneration, and/or inhibit epigenetic modifiers like histone deacetylases (HDACs) to open locked chromatin regions required for structural tract remodelling. On the cellular replacement aspect of adult neurogenesis, these same intracellular interactions can orchestrate the microenvironmental signalling cascades necessary to drive neurogenesis by upregulating intrinsic neurogenic determinants like Wnt/β-catenin and proneural bHLH, among others. As a result, they stimulate neural progenitor proliferation, direct neuroblast migration, and accelerate functional integration into host tissue.

The broad dual-track (axonal repair and neurogenesis) network profile of NPs is however constrained by two major translational challenges: pharmacokinetic (PK) accessibility and intrinsic regenerative efficacy. For far too long, the neuroregeneration field of NPs has focused predominantly on issues of bioavailability, BBB penetration, metabolic stability, and tissue exposure, often treating inadequate delivery as the principal explanation for clinical failure. While these PK barriers remain important, such emphasis risks overlooking a more fundamental question of whether many NPs possess sufficient intrinsic potency/efficacy to drive the magnitude, duration, and coordination of signalling required for meaningful neural repair, even when adequate target exposure is achieved. The following sections therefore examine the dimensions of this problem by giving more emphasis to the limitations associated with the intrinsic regenerative efficacy of NPs.

## 2. The Convergence Paradox and the Bioavailability Fallacy

### 2.1. Structural Diversity Converging to Functional Identity

The diffuse, low-affinity nature of NPs gives rise to a profound pharmacological phenomenon of structural diversity converging to functional identity. When evaluated within controlled cell culture environments (in vitro), molecules featuring completely mismatched chemical skeletons, such as linear plant diphenylheptanoids (e.g., curcumin), polycyclic triterpenoid glycosides (e.g., ginsenosides), and marine-derived polyunsaturated fatty acids, all drain into identical neurogenic outcomes. They all demonstrate the ability to quiet microglial activation, clear reactive oxygen species (ROS), upregulate neurotrophic transcription, and prompt neural stem cells to differentiate into mature neurons. However, when they are transitioned from the two-dimensional setting of cell culture plates to the complex, living three-dimensional environment of an in vivo organism, they showed activity but consistently fail to achieve the required level of functional neuroregeneration needed for clinical outcomes.

### 2.2. When Delivery Is Not Enough

It is important to acknowledge that PK research has delivered substantial advances for neuroactive NPs. Over the past several decades, sophisticated formulation strategies, including nanoparticles, liposomes, polymeric carriers, micellar systems, and intranasal delivery platforms, have significantly improved the stability, brain exposure, and tissue accessibility of numerous NP scaffolds. Likewise, PK optimisation remains an active area of investigation for neurotrophin-based therapeutics, where considerable effort continues to be invested in overcoming the inherent delivery limitations of large protein-based growth factors [38,39]. Despite these important technological achievements, however, the translation of either NPs or neurotrophins into clinically transformative neuroregenerative therapies has remained limited.

Experimental spinal cord injury (SCI) studies indicate that neurotrophins such as BDNF and NGF retain robust biological activity even when delivered directly into the CNS through controlled gradients, biomaterial scaffolds, or cell-based transplantation systems. Neurotrophin gradients can reliably direct axonal extension and orient neurite growth within defined microenvironments, producing spatially organised sprouting responses in vitro and in lesion-adjacent regions [39]. Similarly, Schwann cell transplantation strategies combined with trophic support promote axonal regrowth, remyelination, and partial structural repair within and around injury sites [40]. However, despite these encouraging biological and histological effects, broader meta-analyses of preclinical and clinical SCI research consistently highlight a persistent translational gap, where robust axonal sprouting and local circuit remodelling rarely culminate in long-distance axonal reconnection or durable functional recovery of disrupted sensorimotor pathways [41]. Hence, the clinical studies cited herein provide evidence of safety, feasibility, PKs, or biological activity instead of clinically demonstrated neuroregenerative efficacy. Altogether, these findings suggest that, while neurotrophin-based interventions can initiate and guide local regenerative processes, they are insufficient on their own to achieve the coordinated, large-scale reconstruction required for complete functional restoration of the injured spinal cord. These observations further suggest that successful tissue exposure, while necessary, is not synonymous with successful regeneration. When this principle extends beyond neurotrophin-based therapeutics to the broader class of NPs, improved CNS delivery and target engagement do not consistently translate into durable structural or functional repair. Accordingly, the central limitation may not lie solely in PK accessibility, but in the intrinsic regenerative efficacy of the molecular scaffolds themselves.

It is also essential to distinguish neuroregeneration from neuroprotection, while recognising that many NPs can engage elements of both processes. A substantial body of experimental evidence indicates that NPs may reduce oxidative stress, attenuate inflammation, preserve neuronal viability, and improve functional outcomes in injury and disease models, reflecting robust neuroprotective activity. In parallel, numerous compounds have also been reported to promote neurite outgrowth, axonal sprouting, and neurogenic responses, suggesting engagement of regenerative-associated signalling programmes. These two functional outcomes are not simply different magnitudes of the same response but are often mediated through distinct mechanistic routes. Neuroprotective effects frequently arise from modulation or suppression of injurious pathways such as excessive inflammatory signalling, oxidative stress cascades, or excitotoxic processes, whereas regeneration-associated effects more commonly involve activation or potentiation of growth-related pathways governing cytoskeletal dynamics, developmental transcriptional programmes, and trophic signalling networks (See Section 4). However, despite this mechanistic divergence, both outcomes may be simultaneously triggered by pleiotropic NPs acting across multiple targets and temporal phases. Neuroprotection primarily preserves existing cellular and circuit elements, whereas neuroregeneration requires the coordinated execution of prolonged developmental programmes involving stem-cell activation, axonal extension, target recognition, synaptic integration, and circuit stabilisation. Consequently, the central question is not whether NPs exert biological activity in the injured CNS, which is already well established, but whether their intrinsic regenerative efficacy is sufficient to sustain the complex, temporally organised processes required for durable structural and functional reconstruction. This distinction shifts attention from PK accessibility toward a more fundamental examination of regenerative potency/efficacy.

### 2.3. The Intrinsic Biophysical Reality of NPs

Having established that CNS exposure alone is insufficient to guarantee neuroregenerative efficacy, the limitations of neuroactive NPs must be reconsidered at a more fundamental level. Even when PK constraints are removed and compounds gain direct access to the CNS, their ability to induce durable structural repair of clinical significance remains inconsistent. This suggests that the principal bottleneck is not ligand delivery or receptor engagement per se, but the inability of small-molecule scaffolds to reproduce the integrated, temporally sustained, and spatially coordinated signalling architectures required for developmental-scale tissue reconstruction. Accordingly, the intrinsic biophysical limitations of NPs can be conceptualised across three interrelated dimensions: receptor-level modulation dynamics, stage-dependent signalling incompatibility, and regenerative network incompleteness.

#### 2.3.1. Insufficient Receptor Engagement for Sustained Signalling

The first component of this triad is insufficient receptor engagement for sustained signalling. In the context of endogenous neurotrophin biology, BDNF promotes long-distance axonal extension and induces neural stem cell differentiation because it is a large, highly ordered homodimeric protein. Its precise spatial architecture enables it to bridge two separate full-length TrkB (TrkB.FL) receptor monomers, stabilising an active receptor dimer and sustaining the prolonged receptor autophosphorylation required to execute complex transcriptional and cytoskeletal programmes over hours to days [42,43,44]. By contrast, the majority of single neuroactive NPs, including plant flavonoids, fungal tryptamines, and marine-derived fatty acids, do not primarily function through stable receptor engagement. Instead, their biological effects are often mediated through indirect modulation of intracellular signalling networks, stress-response pathways, or upstream regulatory targets (Section 4), with only a subset demonstrating direct receptor-level activity. Even in cases where receptor engagement is reported, such interactions are typically characterised by comparatively transient and lower-avidity binding dynamics that are insufficient to sustain long-duration signalling states.

The relatively weaker receptor engagement limitation of NPs is exemplified by 7,8-dihydroxyflavone (7,8-DHF), a natural flavone widely investigated as a small-molecule TrkB agonist [45]. While 7,8-DHF induces acute activation of downstream Akt and ERK signalling pathways in vitro, evidence suggests that its receptor engagement does not fully reproduce the spatial, temporal, and signalling characteristics of endogenous BDNF [46]. Owing to its small molecular size, limited structural rigidity, and comparatively short receptor engagement dynamics, 7,8-DHF is unlikely to sustain the prolonged receptor activation, trafficking behaviour, and transcriptional programmes associated with native neurotrophin signalling. Consequently, while sufficient to initiate early regenerative signalling cascades such as transient ERK/MAPK and PI3K/Akt activation, such interactions may not fully support the extended CREB-dependent transcriptional programmes, metabolic reprogramming, and cytoskeletal remodelling required for robust axonal elongation and durable neural stem/progenitor cell responses [46]. Nevertheless, 7,8-DHF remains one of the best-characterised examples of an NP-derived scaffold capable of directly engaging TrkB signalling, whereas many other neuroregenerative NPs exert their effects indirectly through anti-inflammatory, metabolic, antioxidant, or epigenetic mechanisms, just to mention few examples. The 7,8-DHF therefore represents not a failed neurotrophin mimic but a valuable starting pharmacophore for pseudo-natural product (PNP) discovery, providing a validated structural template that can be incorporated into monovalent or multivalent hybrid architectures designed to enhance receptor residence time, signalling persistence, and regenerative efficacy.

#### 2.3.2. Stage-Dependent Signalling Incompatibility

The second component of the intrinsic biophysical reality of NPs in neuroregeneration is stage-dependent signalling incompatibility. Neuroregeneration is not a singular biological event but a temporally ordered process comprising inflammatory remodelling, neural stem/progenitor cell activation, axonal extension, guidance, synaptic integration, and circuit stabilisation. These phases are governed by signalling systems that are inherently regulated by spatial and temporal dynamics [47]. Following neural injury, an acute inflammatory response is required for debris clearance, extracellular matrix remodelling, and initiation of regenerative programmes, whereas sustained inflammatory signalling promotes glial scar formation and inhibits axonal regrowth [48]. Similarly, ROS signalling can act as an early regenerative signal through redox-sensitive transcriptional programmes including nuclear factor erythroid 2-related factor 2 (Nrf2) activation, but prolonged oxidative stress impairs neuronal survival and axonal extension [49]. Developmental growth-regulating pathways demonstrate the same temporal separation of function. Notch signalling maintains neural stem/progenitor cell pools and prevents premature differentiation, but prolonged Notch activation suppresses neuronal maturation and integration [50]. The mTOR signalling supports axonal growth and protein synthesis during regenerative phases, but sustained activation can lead to metabolic imbalance and aberrant sprouting, as observed in CNS injury models [51]. The ERK signalling similarly regulates plasticity-related transcriptional programmes required for structural remodelling but prolonged activation can decouple growth signalling from coordinated circuit stabilisation. Evidence suggests that while moderate activation supports proliferation, and survival, excessive or insufficient ERK activity inhibits growth [52].

Moreover, the above-mentioned temporal organisation is not unique to injury biology as it also reflects a fundamental property of endogenous neurotrophic signalling systems. Neurotrophins such as BDNF operate through tightly regulated spatial and temporal signalling domains, where receptor activation is coupled to trafficking dynamics, feedback regulation, and activity-dependent expression patterns [53]. In this sense, neurotrophins provide an internal example of how regenerative signalling is naturally constrained by time-structured control mechanisms. On these bases, the limitation associated with many neuroactive NPs arises not from their inability to engage individual pathways, but from their inability to align with these temporally segregated biological requirements. Since most NPs simultaneously modulate multiple signalling targets (e.g., CREB, Akt, ERK, Nrf2, etc.), their effects are distributed across early and late regenerative phases without intrinsic temporal partitioning. This lack of temporal resolution may result in biological activities that are beneficial during one phase of repair becoming neutral or inhibitory in another, thereby reducing the efficiency of progression through the full regenerative sequence. In contrast, neurotrophin systems achieve phase progression through tightly regulated ligand availability, receptor trafficking, and activity-dependent feedback control, enabling sequential execution of regenerative programmes. Therefore, the challenge for neuroactive NPs is not solely pathway activation, but the absence of intrinsic mechanisms that enforce temporal ordering of signalling events. This limitation contributes to the observed disconnect between robust bioactivity in simplified experimental systems and inconsistent translation into durable structural regeneration in vivo. It also provides a mechanistic rationale for PNP design strategies aimed at engineering temporal control into multi-target pharmacology through structurally integrated, modular pharmacophore architectures.

#### 2.3.3. Incomplete Regenerative Network Convergence

The third component of the intrinsic biophysical reality of NPs is related to incomplete regenerative network convergence. Successful CNS repair requires the coordinated engagement of multiple partially independent biological subsystems, including intrinsic axonal growth programmes, cytoskeletal remodelling machinery, mitochondrial metabolic adaptation, axon guidance interpretation systems, synaptic maturation pathways, and inflammatory resolution circuits. Endogenous neurotrophins, particularly BDNF and NGF, represent the closest biological example of integrated regenerative signalling systems. Through high-affinity Trk receptor engagement and tightly regulated spatial signalling domains, NTs propagate coordinated downstream cascades that couple axonal growth, cytoskeletal remodelling, metabolic adaptation, and synaptic maturation into a unified regenerative program. However, even this endogenous system does not fully resolve the challenge of functional CNS regeneration in pathological conditions, as regenerative outcomes remain constrained by injury-induced architectural disruption, receptor downregulation, and inhibitory extracellular environments. Against this benchmark, most neuroactive NPs do not achieve comparable system-level integration (Section 5). Instead, they preferentially modulate a limited subset of regenerative modules, frequently converging on antioxidant responses (e.g., Nrf2-associated signalling), inflammatory regulation (e.g., nuclear factor kappa-light-chain-enhancer of activated B cells (NF-κB) pathways), or secondary neurotrophic mediators (e.g., CREB/Akt/ERK activation). While these effects are biologically reproducible and often robust in experimental models, they remain functionally distributed and may not integrate into a unified regenerative output. The resulting state of partial functional activation in which discrete components of the regenerative machinery are engaged without reaching the threshold of coordinated multi-system output required for long-distance axonal regeneration and stable circuit reconstruction. The translational challenge is then seen not as a failure of NPs pharmacology per se, but as a limitation of its fragmented architecture which may be resolved by employing the PNP strategy.

## 3. The Pseudo-NP Approach

**Figure 3:**
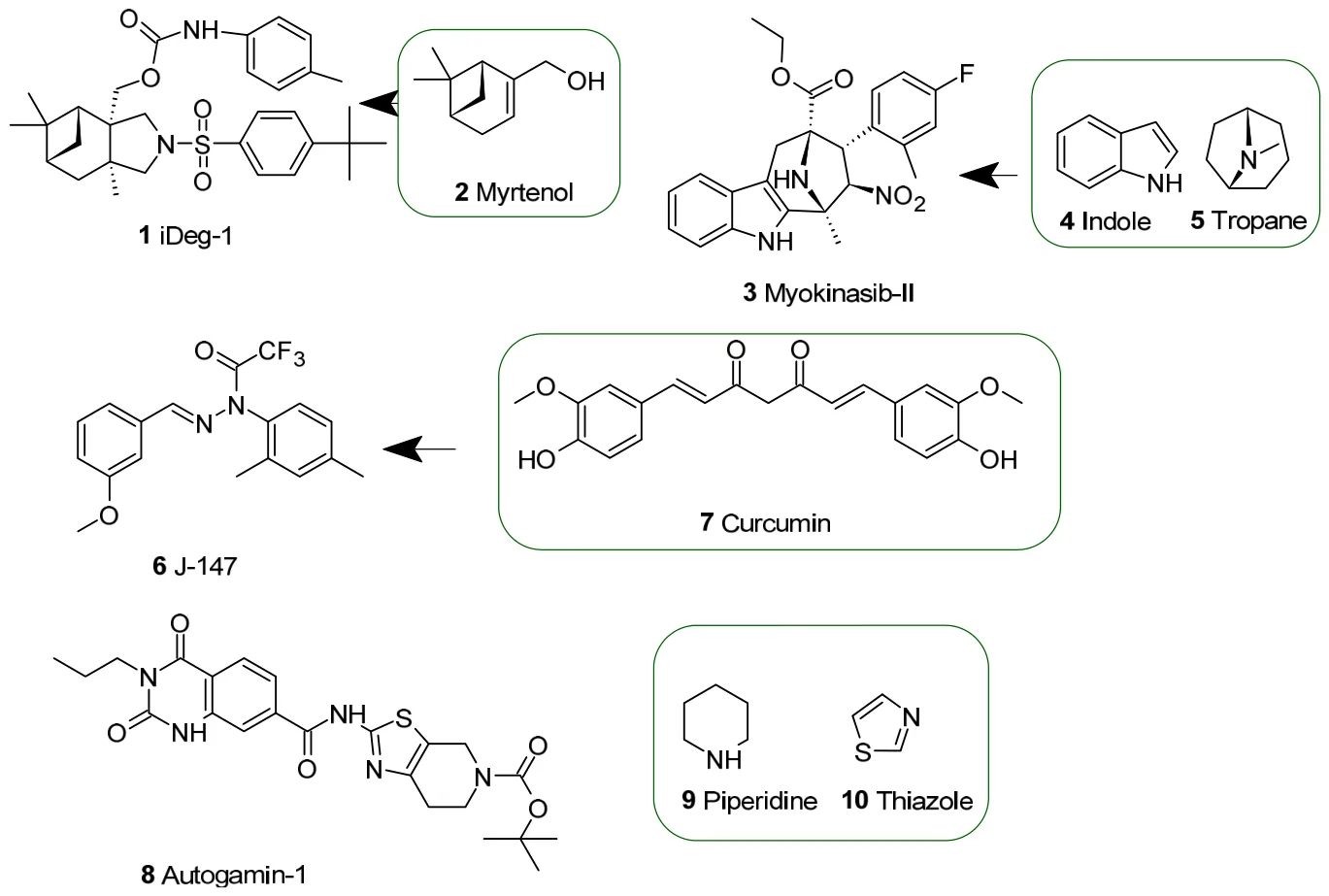
Examples of PNPs. Structural fragments or NPs used for the PNP synthesis strategy are shown in green boxes.

The PNPs represent a deliberate, human-driven chemical evolution in which biosynthetically unrelated natural fragments are computationally deconstructed, stripped of metabolic instability and non-productive cross-talk liabilities, and reassembled into non-natural hybrid scaffolds with emergent functional properties. The application of PNPs in drug discovery has been extensively reviewed [54] and its validity as a robust technology platform is supported by multiple convergent advances in modern medicinal chemistry. A prominent example is the targeted protein degradation approach, which has introduced monovalent PNP architectures such as indoleamine-2,3-dioxygenase 1 (IDO1) degraders (e.g., iDeg-1 (**1**), Figure 3), derived from natural scaffolds including (−)-myrtanol (**2**) [55]. The identification of this chemotype originated from high-throughput screening of a 157,332-compound library, underscoring the scale at which NP-derived chemical space can be reorganised into functionally active synthetic architectures. Instead of operating as classical heterobifunctional degraders that depend on artificial recruitment of external E3 ligases, these hybrid molecules act by destabilising the IDO1 structure through displacement of the haem cofactor, thereby promoting protein degradation via endogenous proteostatic machinery, specifically the cullin-RING E3 ligase pathway. This combined mechanism of functional inhibition and induced protein destabilisation enables simultaneous suppression of both enzymatic activity and non-enzymatic signalling functions of IDO1 in inflammatory and oncological diseases.

Another complementary example is provided by autogramin design (e.g., autogramin-1 (**8**) (Figure 3), in which fusion of aliphatic piperidine (**9**) and thiazole (**10**) fragments generates selective ligands targeting the steroidogenic acute regulatory protein-related lipid transfer domain of GRAM domain containing 1A [56]. By directly competing with cholesterol binding, autogramins act as precise molecular probes of lipid-dependent regulation of starvation-induced autophagosome biogenesis, thereby revealing mechanistic checkpoints in macroautophagy control. The broader validity of the PNP approach is further supported by large-scale cheminformatic analyses. Mapping of the ChEMBL database indicates that PNP-like architectures represent a substantial proportion of known bioactive compounds, and expands further among recent compounds reaching clinical trials [57]. Within CNS therapeutics, early examples also demonstrate how PNP principles can extend beyond conventional PK optimisation and address intrinsic limitations of NP pharmacology. The J147 (CNB-001 (**6**), Figure 3), as a highly potent and orally active synthetic derivative of curcumin (**7**), provides a key proof-of-concept [58,59]. In this design, the metabolically unstable and pan-assay-interfering β-diketone core of curcumin was removed while preserving key feruloyl/*O*-methoxyphenol pharmacophoric elements. These were integrated into a rigid synthetic cyclohexyl-pyrazole scaffold to allow a structurally stable and pharmacologically refined molecule. This structural reconfiguration is accompanied by a marked shift in potency where the promiscuous low-affinity, multi-target interactions in the micromolar (μM) range are converted to J147 with high-affinity activity at low-nanomolar (nM) concentrations (EC_50_ 25 nM) [60,61]. This increase in target precision is associated with selective interaction with the mitochondrial protein ATP synthase subunit α (ATP5A), positioning mitochondrial bioenergetic regulation as a primary upstream target. Through this interaction, J147 modulates downstream CAMKK2-AMPK-mTOR (calcium/calmodulin-dependent protein kinase kinase 2-activating AMP-activated protein kinase-mechanistic target of rapamycin) signalling pathways, which are linked to enhanced neurotrophic signalling, including BDNF and NGF expression. Functionally, this mechanistic shift is associated with improved neurogenic and regenerative phenotypes in preclinical CNS models. Its progression into early clinical trials [62] illustrates that a central principle of PNP design to overcome intrinsic potency and selectivity limitations of NPs is far better than relying on formulation or delivery-based optimisation alone. Simultaneously, indotropane-based PNP compounds (e.g., myokinasib II (**3**), Figure 3) illustrate how NP-derived privileged scaffolds can be recombined across biosynthetic boundaries to generate highly selective modulators of discrete signalling targets [63]. These constructs function as precision chemical probes capable of modulating specific kinase-dependent pathways implicated in inflammation, infection persistence, and neuroimmune regulation in preclinical systems. Hence, experimental evidence so far showed that PNPs, as a human-driven chemical evolution for discovering new chemotypes and unexpected bioactivities in drug discovery [64], can be applied in neuroregeneration.

## 4. Decoding the Natural Products Goldmine: A Taxonomical Audit of Structural Convergence

### 4.1. Methodological Landscape Based on Evidence from Fifty Years of Neuroregenerative Research

A structured literature search was undertaken to identify NPs investigated experimentally for neuroregeneration from the earliest relevant literature through to the current (until August 2026) literature. Searches were conducted in PubMed, ScienceDirect, and Google Scholar, supplemented by backward and forward citation tracking of key publications. The objective was not designed to catalogue all NPs with reported effect on neuronal biology, but to identify compounds repeatedly represented in the experimental literature and subsequently select a mechanistically diverse set of representative compounds across chemical classes of NPs, biological sources, regenerative phenotypes, and molecular targets.

The search strategy combined four concept domains using combinations of terms as follows: natural product/source, regenerative phenotype, experimental model/injury, and molecular mechanism. Natural-product terms included natural product, natural compound, bioactive compound, phytochemical, secondary metabolite, plant-derived, fungal metabolite, mushroom-derived, microbial metabolite, and marine natural product, along with major structural classes including flavonoids, polyphenols, phenolics, terpenoids, terpenes, alkaloids, polyketides, quinones, xanthones, coumarins, lignans, saponins and related classes. Neuroregenerative terms included neuroregeneration, neural regeneration, neuronal regeneration, axon/axonal regeneration, axon/axonal growth, neurite outgrowth, neurite extension, neuronal sprouting, nerve regeneration, neurogenesis, adult neurogenesis, hippocampal neurogenesis, neural stem/progenitor cells, and neuronal differentiation. These were combined with terms describing the principal experimental contexts, including spinal cord injury, traumatic brain injury, ischemic stroke, cerebral ischemia, intracerebral haemorrhage, peripheral nerve injury (PNI), sciatic nerve injury, nerve crush, and nerve transection, as well as relevant cellular systems such as primary cortical or hippocampal neurons, pheochromocytoma (PC12) cells, neuroblastoma (SH-SY5Y, astrocytes, microglia, neural stem/progenitor cells and co-culture models.

Since neuroregeneration terminologies have changed substantially over the past five decades, phenotype-based terms such as neurite extension, axonal growth, neuronal differentiation, and nerve repair were deliberately included. Candidate compounds identified through these searches were subsequently searched individually using their names and synonyms in combination with regenerative phenotypes, injury models, cellular systems and reported molecular targets or signalling pathways. Only original experimental research articles were considered as evidence for compound selection. Review articles, systematic reviews, meta-analyses and other secondary publications were excluded from the evidence dataset, although their reference lists could be used to identify additional original studies. Experimental evidence was required to demonstrate a regenerative phenotype or a mechanism directly associated with regeneration. Neuroprotection alone, including reductions in oxidative stress, inflammation, apoptosis, excitotoxicity or neuronal loss, was not considered sufficient unless accompanied by evidence of axonal recovery, neurite/axon outgrowth, nerve regeneration, adult neurogenesis, neuronal replacement/differentiation, or another defined regenerative outcome. Similarly, changes in regenerative markers or signalling proteins without an associated experimental phenotype were not independently classified as regenerative evidence.

**Figure 4:**
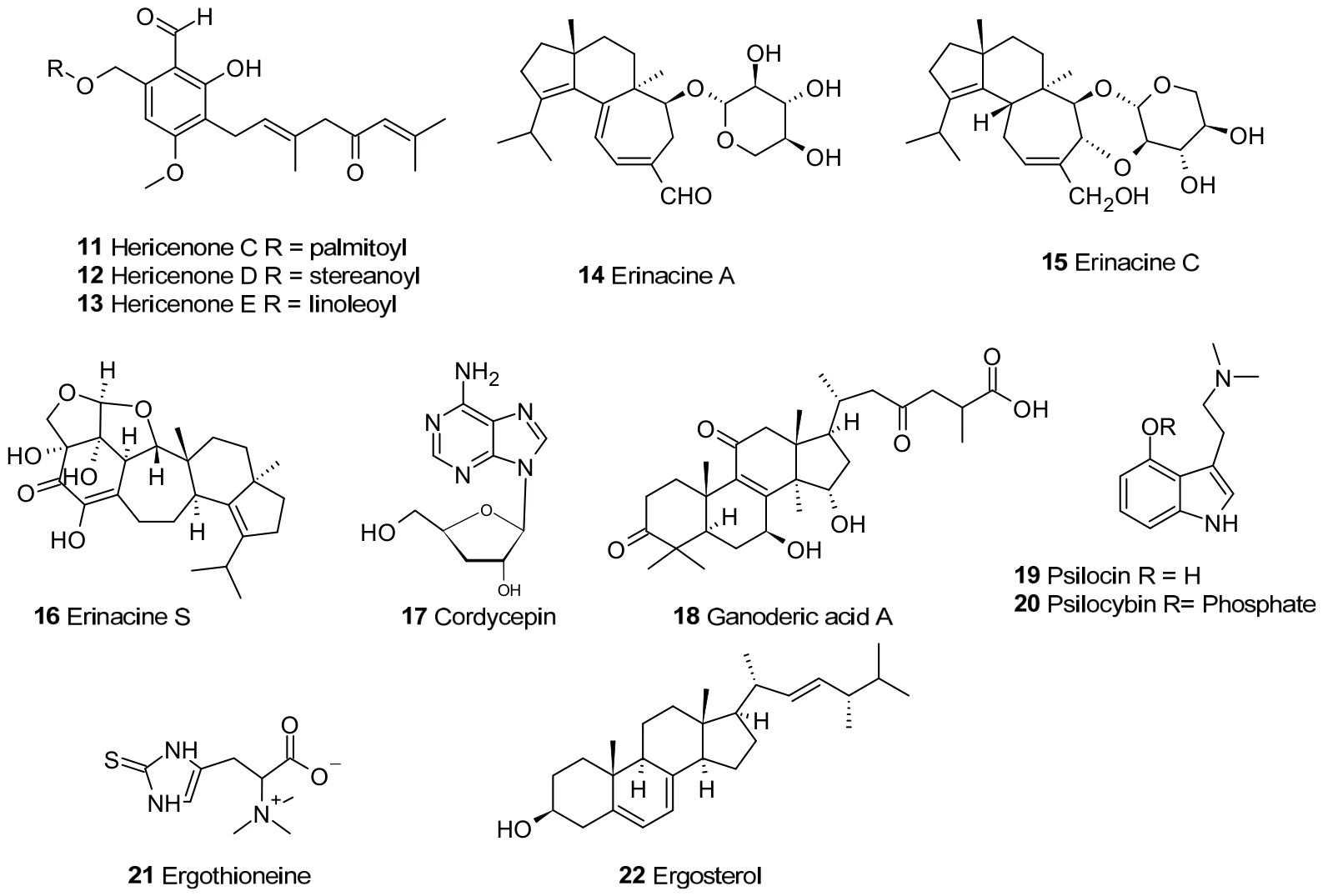
Representative mushroom-derived compounds in neuroregeneration research.

**Figure 5:**
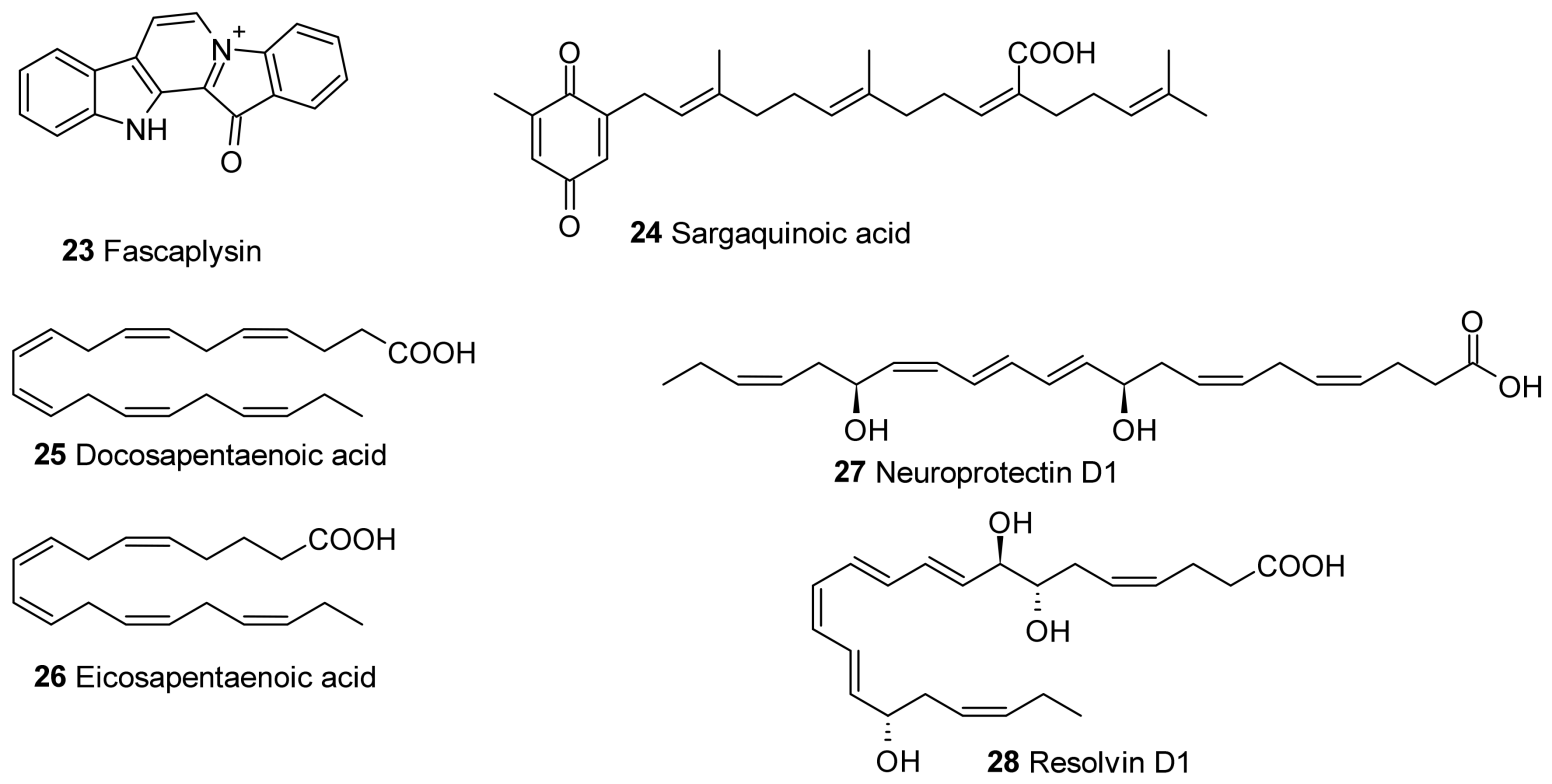
Example of some marine-derived biomolecules in neuroregeneration research.

**Figure 6:**
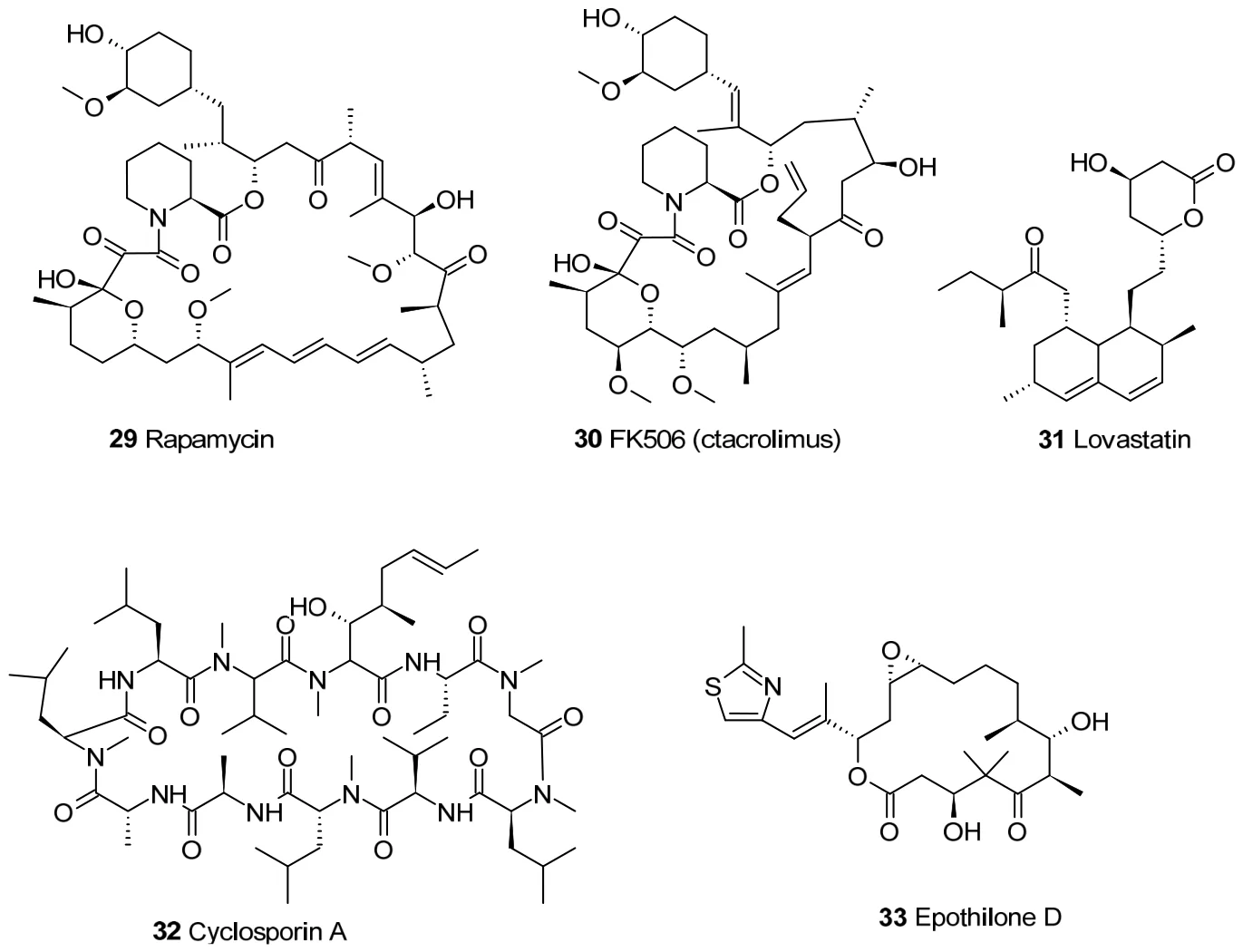
Representative microbial-derived natural products in neuroregeneration research.

**Figure 7:**
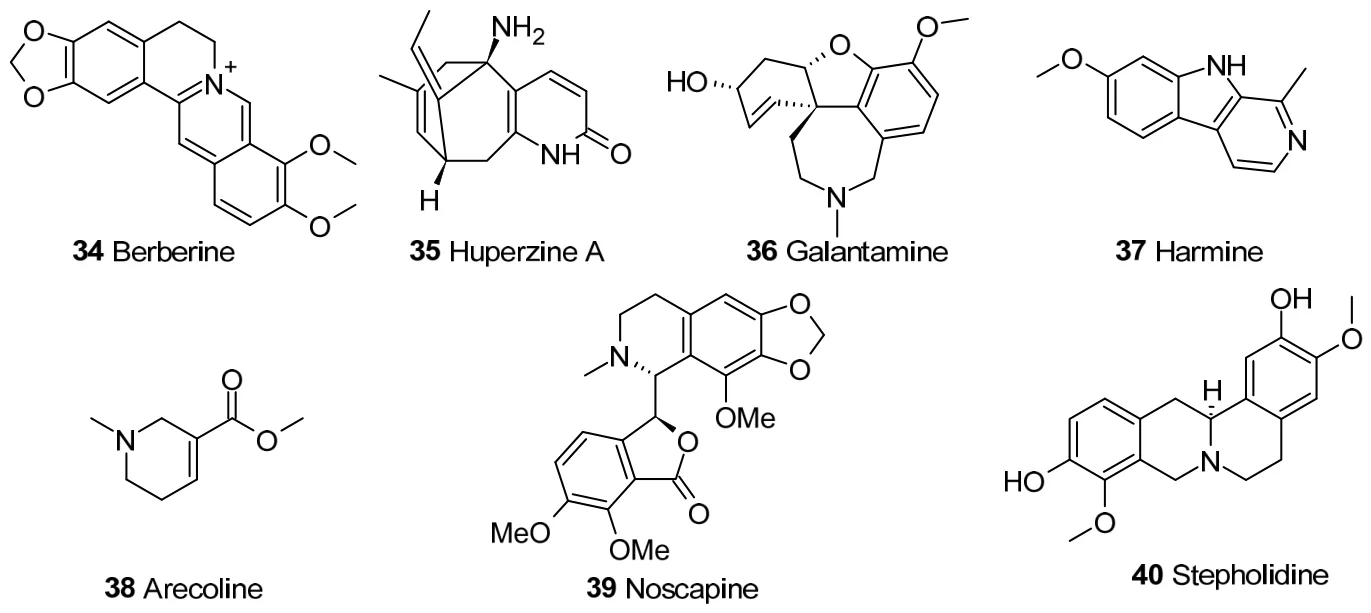
Structures of representative alkaloids of plant origin in neuroregeneration research.

**Figure 8:**
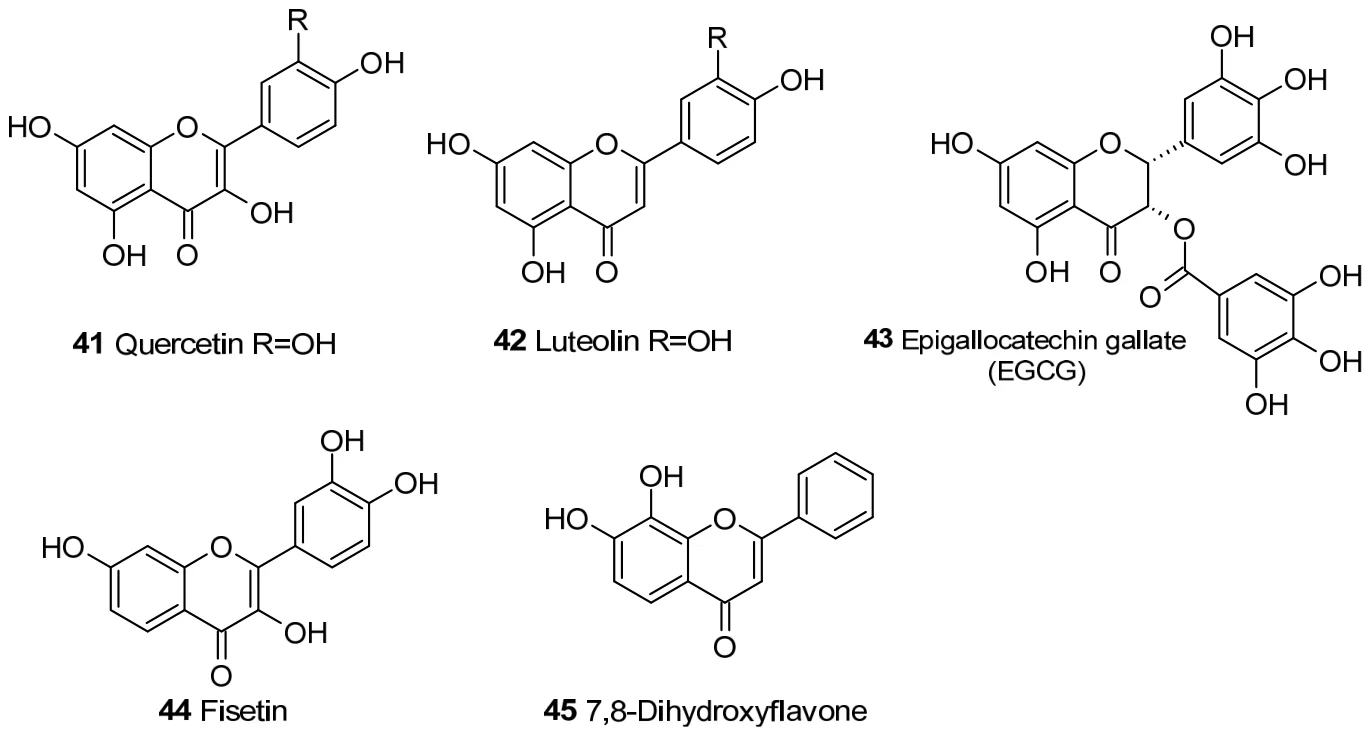
Structures of some flavonoids in neuroregeneration research.

**Figure 9:**
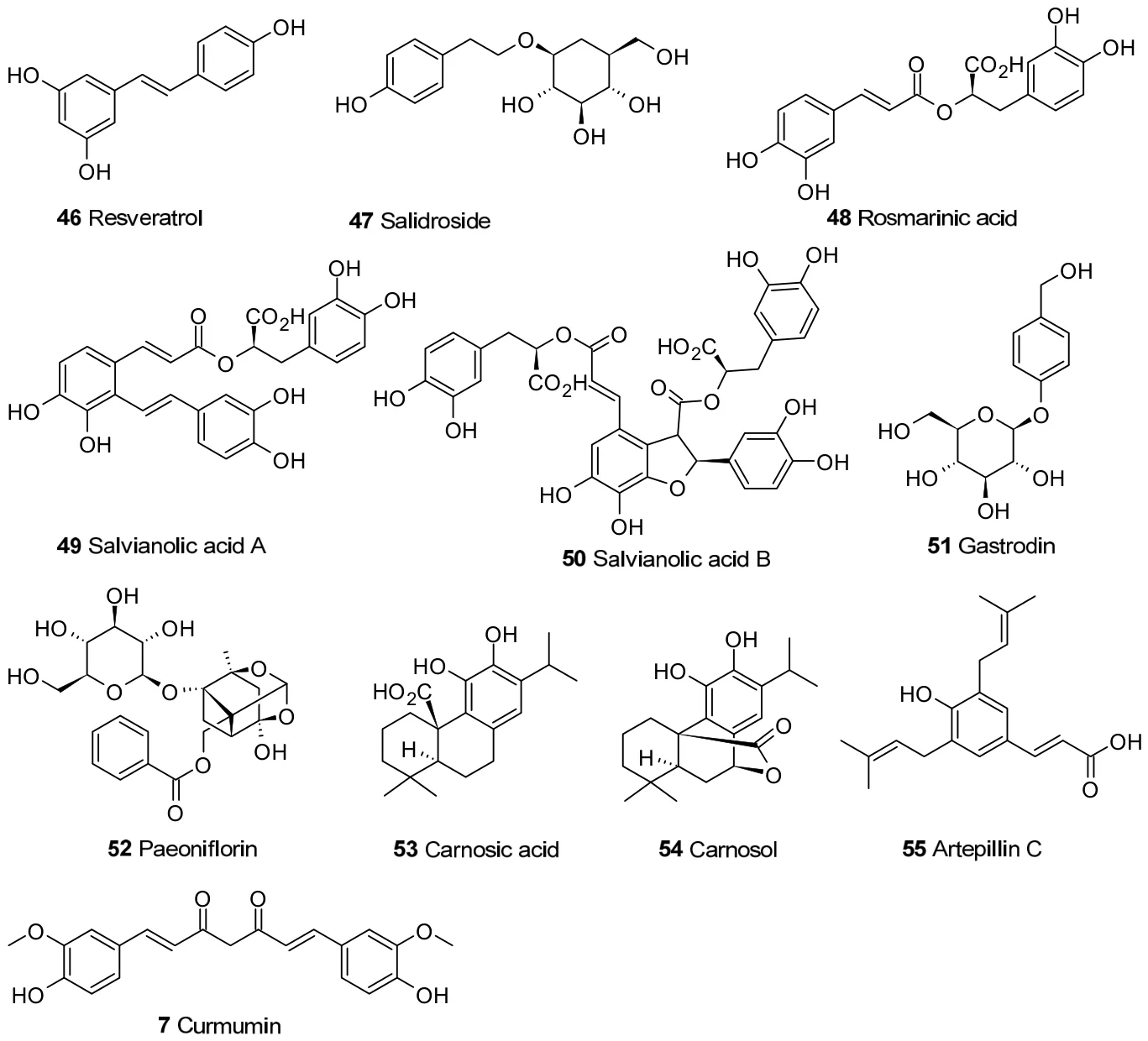
Representative polyphenols other than flavonoids in neuroregeneration research.

**Figure 10:**
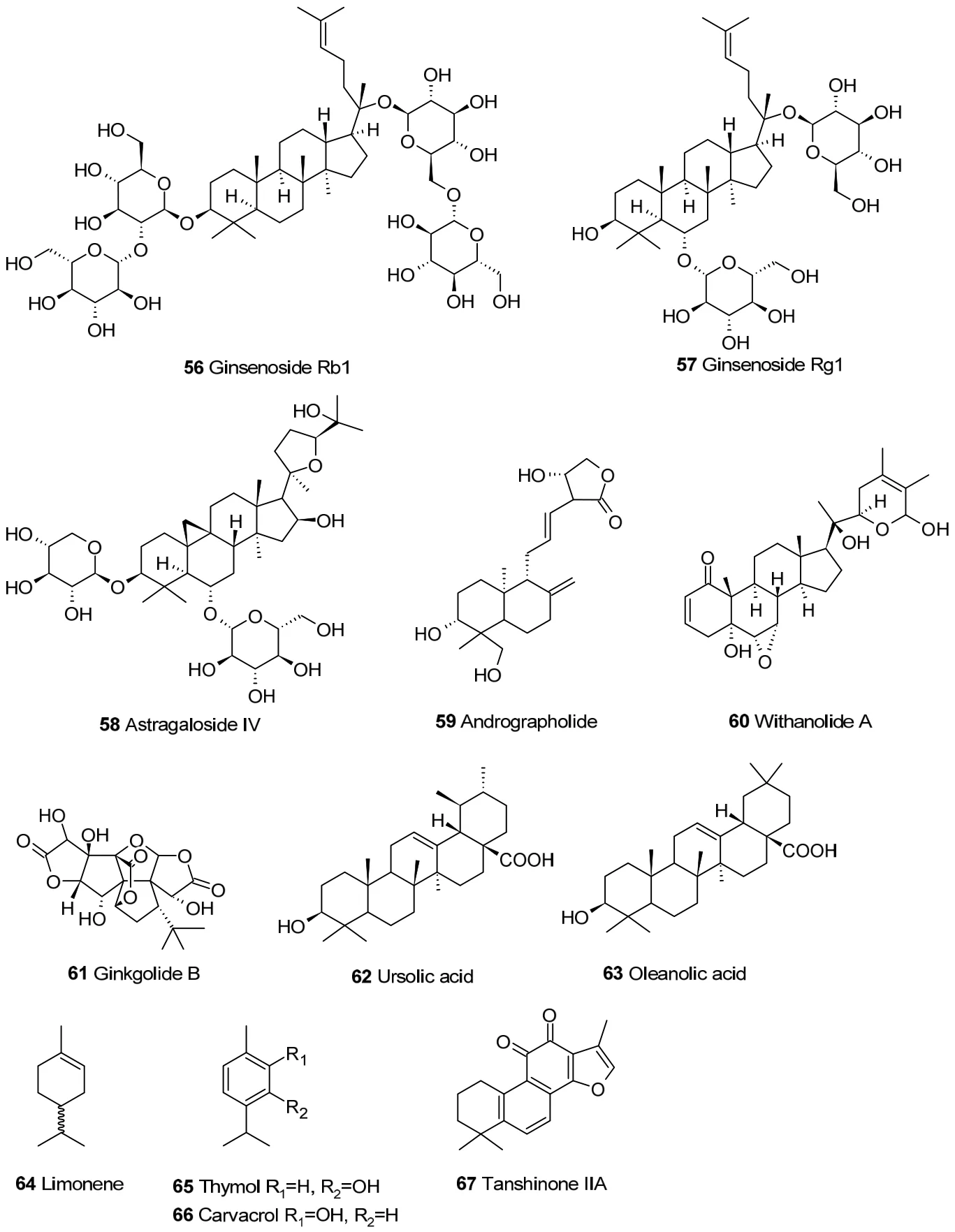
Examples of terpenoids of plant origin in neuroregeneration research.

**Figure 11:**
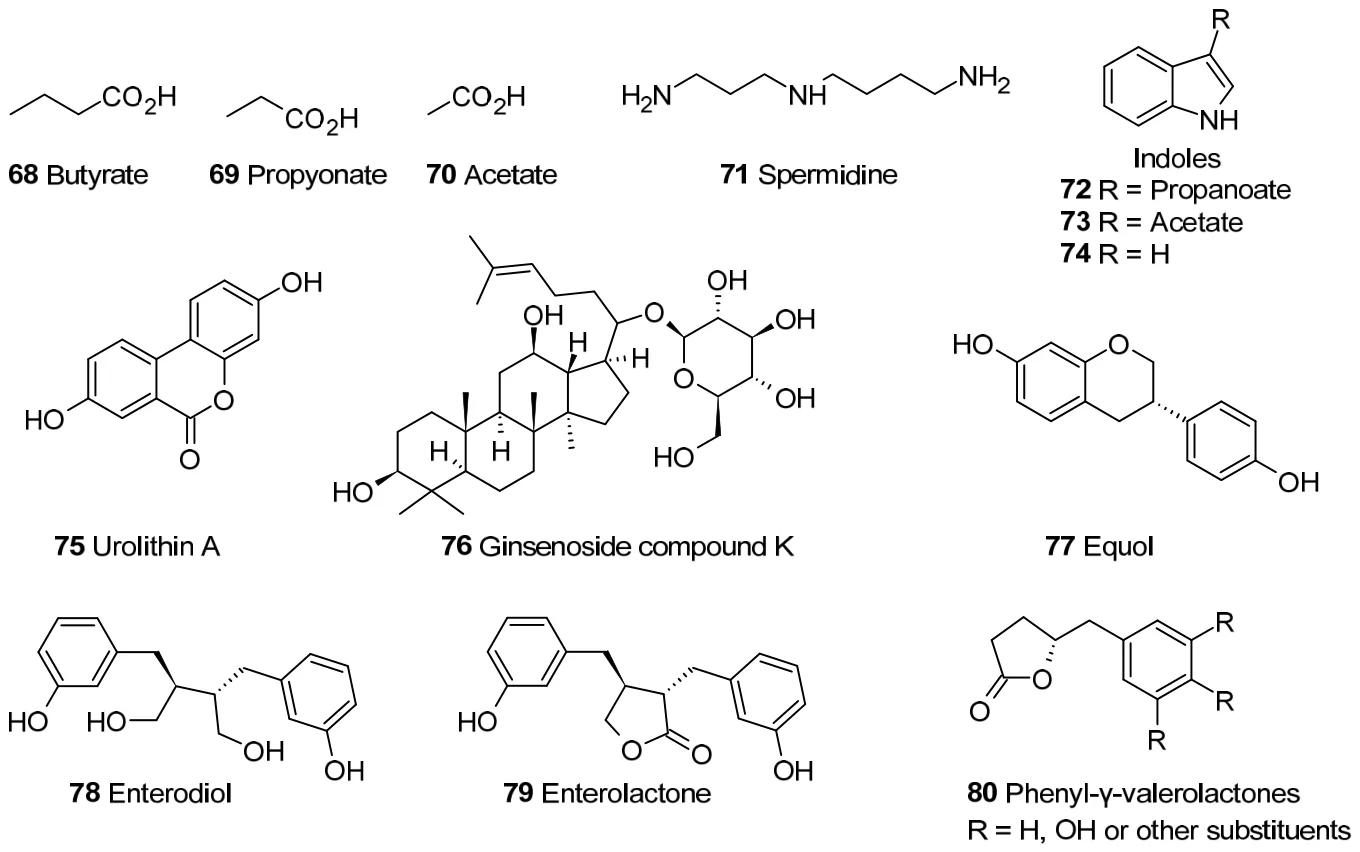
Gut microbiota-derived metabolites of natural products in neuroregeneration research. The gut microbiota-derived NPs in neuroregeneration include short chain fatty acids (SCFAs) including butyrate, propionate and acetate, indoles such as derivatives of propionate or acetate and spermidine. Urolithin A as exemplary urolithins obtained from ellagitannins and ellagic acid and ginsenoside compound K from microbial processing ginsenosides are among other examples.

Following identification of candidate compounds, the literature was organised by chemical class and biological source. Repeated occurrence across independent original studies was used to establish recurrence within the literature, but publication frequency was not used as an indicator for therapeutic efficacy or as the sole criterion for selection. Compounds were selected to provide complementary representation of chemical diversity and experimentally supported mechanisms. These include alkaloids, terpenoids, flavonoids, and non-flavonoid polyphenols derived from plant, fungal, microbial, and marine sources (Figure 4, Figure 5, Figure 6, Figure 7, Figure 8, Figure 9, Figure 10 and Figure 11), each demonstrating reproducible bioactivity across multiple neuroregeneration-related experimental systems. Closely related compounds or members of large metabolite families were therefore not exhaustively enumerated. For example, the numerous hericenones and erinacins reported from mushrooms were represented by a limited number of compounds selected to capture distinct mechanistic or experimental dimensions, and the same principle was applied to other structurally related NP families.

Final inclusion was therefore mechanism-stratified instead of frequency-based. When several compounds produced a similar regenerative phenotype, preference was given to those providing experimentally supported differences in molecular targets, signalling pathways, cellular mechanisms, injury models, in vivo evidence, or translational relevance. Thus, enhancement of neurogenesis or neurite outgrowth alone did not determine inclusion where multiple compounds showed comparable phenotypes; compounds offering distinct mechanistic information were prioritised. For each selected compound, information was extracted on natural source, structural class, experimental model, injury paradigm, regenerative endpoint, molecular target(s), signalling pathway(s), cellular mechanism, in vivo evidence, and available pharmacokinetic or bioavailability information. The approximately 80 resulting compounds (Figure 4, Figure 5, Figure 6, Figure 7, Figure 8, Figure 9, Figure 10 and Figure 11) were consequently treated as a representative mechanistic landscape of NP neuroregeneration which is by no means an exhaustive catalogue. The selection process followed the sequence: broad literature search → identification of candidate compounds → verification using original experimental studies → exclusion of non-regenerative or insufficiently supported evidence → chemical-family grouping → mechanistic clustering → removal of redundancy → selection of representative compounds. This approach enabled approximately five decades of heterogeneous literature to be integrated while preserving both chemical diversity and mechanistic distinctiveness. Altogether, this body of studies reveals a recurring pattern in which structurally diverse NPs converge on overlapping functional domains of neuroregenerative biology. This dataset can be interpreted as a distributed map of bioactivity space in which chemically unrelated scaffolds influence shared injury-associated cellular states and regenerative processes.

Moreover, this review does not imply unique scaffold–function relationships. It only highlights functional convergence across chemically heterogeneous compounds within complex biological systems. From this viewpoint, the longstanding NPs literature may be interpreted not as a catalogue of finalised regenerative therapeutic solutions, but as a pre-organised and functionally annotated fragment space. Within this scope, a PNP strategy can be viewed as an approach for systematically interrogating and recombining these validated bioactive NP fragments to explore whether a far more potent and efficacious compound can be designed for neuroregenerative function.

### 4.2. Upstream Biochemical Permissiveness: Clearing the Injured Microenvironment

The first chronological gate of in vivo neuroregeneration involves the transition of the injured CNS from a hostile, pro-inflammatory, and oxidatively stressed environment into a biochemically permissive niche capable of supporting neuronal survival and subsequent axonal growth. Across primary microglia and astrocyte cultures, as well as rodent models of TBI, SCI, and stroke, a broad range of chemically diverse NPs converge on modulation of two central redox- and inflammation-sensitive signalling axes: the Nrf2 antioxidant system and the NF-κB inflammatory pathway. Activation or modulation of the Nrf2 pathway is consistently associated with induction of endogenous antioxidant defence systems, including upregulation of superoxide dismutase (SOD), haeme oxygenase-1 (HO-1), glutathione biosynthesis, and phase II detoxification enzymes. This reduces oxidative burden and restores redox balance within injured neural tissue, thereby establishing conditions permissive for regeneration. This functional convergence is observed across structurally diverse NP classes, including plant stilbenes and phenolic acids (e.g., resveratrol (**46**), salvianolic acids (**49**,**50**), and rosmarinic acid (**48**)), phenolic (e.g., carvacrol, (**66**), thymol, (**65**)) or nonphenolic (e.g., limonene, (**64**)) monoterpenes, mushroom-derived compounds (e.g., ergothioneine (**21**), and ganoderic acid A (**18**)), flavonoids such as flavones (e.g., luteolin, (**42**)), flavanols (e.g., quercetin, (**41**)) or flavans (e.g., epigallocatechin gallate (EGCG, (**43**))), and terpenoid glycosides (e.g., ginsenosides (**56**,**57**)).

Among flavonoids, luteolin (**42**) has been shown in hippocampal and TBI models to simultaneously enhance neurotrophic signalling (BDNF, NT-3, TrkB) and activate the Nrf2 antioxidant pathway [65,66]. Mechanistically, luteolin promotes nuclear translocation of Nrf2 and increases downstream antioxidant gene expression including HO-1 and NQO1, with loss of effect in Nrf2-deficient mice confirming pathway dependence. In a rat model of spinal cord ischemia-reperfusion injury, similar Nrf2-dependent antioxidant and anti-inflammatory effects were observed, reinforcing its dual role in neuroprotection and neuroregeneration [67]. Resveratrol (**46**) similarly promotes recovery in SCI models through modulation of the Nrf2/HO-1 axis while also enhancing autophagy-related pathways (increase LC3-II and Beclin-1 levels), linking redox regulation with cellular clearance mechanisms [68,69]. The Nrf2 activation by resveratrol is additionally implicated in spinal cord dorsal column hypoxia models, further supporting its conserved role across injury contexts [70]. Other phenolic compounds such as salvianolic acid A (**49**) similarly activate Nrf2 signalling in ischemic stroke models by increasing Nrf2 nuclear activity, suppressing Keap1, and upregulating downstream antioxidant enzymes (NQO1, SOD1, SOD2), while restoring glutathione (GSH) metabolism (GSH, xCT, GPX4 (glutathione peroxidase-4)) [71]. Gastrodin (**51**) also enhances Nrf2/HO-1 signalling in N2a/APP cellular models, leading to improved synaptic function and reduced oxidative injury [72]. Beyond phenolics, non-phenolic compounds such as ginsenoside Rb1 (**56**) and andrographolide (**59**) similarly activate Nrf2/HO-1 signalling to reduce oxidative stress and neuronal injury in neural progenitor cells and subarachnoid haemorrhage models respectively [73].

Similarly, suppression of the NF-κB inflammatory axis represents a second major mechanism by which NPs establish a permissive regenerative environment. Inhibition of NF-κB activation reduces transcription of pro-inflammatory cytokines including interleukin (IL)-1β, IL-6, and tumour necrosis factor-α (TNF-α), thereby limiting secondary injury, glial activation, and chronic neuroinflammation. Gastrodin (**51**) exerts anti-inflammatory effects through modulation of NF-κB and downstream cytokines (IL-1β, IL-6, TNF-α), leading to improved neurogenesis and synaptic protection in both amyloid and injury models [72]. Paeoniflorin (**52**) also improves functional recovery after ischemic stroke by inhibiting NF-κB signalling and suppressing pro-inflammatory cytokine production, thereby facilitating neurogenesis and repair [74,75]. Similarly, salvianolic acid B (**50**) suppresses NF-κB activation and reduces inflammatory cytokine release (IL-1β, IL-6, TNF-α) in TBI models, thereby reducing neuroinflammation and improving functional outcomes [76]. Likewise, thymol (**65**) suppresses microglial activation and attenuates production of pro-inflammatory mediators in ischemic brain injury models [77], while carvacrol (**66**) exerts similar neuroprotective effects in diffuse TBI through downregulation of NF-κB signalling and reduction of IL-1β, IL-6, and TNF-α expression [78]. Although the phenolic structural moiety as shown in these simple monoterpene-based compounds is often linked to their antioxidant and anti-inflammatory properties, insight from research on limonene (**64**) gives a different perspective. In SH-SY5Y neuroblastoma and BV-2 microglia co-culture, (+)-limonene attenuated the microglia-mediated inflammation by downregulating the secretion of pro-inflammatory cytokines. In oxidative stress condition induced by experimental agents, it also reduced the level of ROS and increased antioxidant defences including GPx and SOD activity [79]. The anti-inflammatory activity of limonene including suppression of IL-1β and TNF-α in an experimental model of neuroregeneration as shown in sensory and motor function recovery was coupled with several biochemical improvements and outcomes [80].

Urolithin A (**75**) inhibits NF-κB signalling while also suppressing the NLRP3 (NOD-like receptor family pyrin domain containing 3) inflammasome activation, leading to a reduction in inflammatory secondary injury and promotion of axonal regeneration in PNI damage [81,82,83]. Urolithin A also alleviates BBB disruption and attenuates neuronal apoptosis following TBI in mice by suppressing NF-κB signalling and reducing pro-inflammatory cytokine production [83]. Andrographolide similarly preserves BBB integrity following acute brain injury through inhibition of NF-κB-associated inflammatory cascades and reduction of vascular permeability [84]. In addition, curcumin (**7**) enhances neurological recovery and neurogenesis following SCI by suppressing the NLRP3 inflammasome pathway, leading to reduced IL-1β and IL-18 signalling and attenuation of neuroinflammatory cascades [85].

As a structural group, polyunsaturated fatty acids (PUFAs) such as docosahexaenoic acid (DHA, (**25**)) and eicosapentaenoic acid (EPA, (**26**)) are well characterised in neuroregeneration, where their functional benefit was shown to be associated with anti-inflammatory mechanisms such as reduction of proinflammatory cytokines [86]. Even pilot clinical study in patients with type 1 diabetes showed that low plasma DHA level was associated with prevalent distal-symmetric-polyneuropathy, and therapy with PUFAs to baseline blood level was associated with greater nerve regeneration [87]. The enzymatically derived derivatives of DHA products in neuronal cells as anti-inflammatory and survival factors are exemplified by neuroprotectin D1 (**27**) and resolvin D1 (**28**). They promote neurogenesis and angiogenesis, BBB integrity in an experimental ischemic stroke model [88] and SCI [89] models.

The peripheral nerve regeneration effect FK506 (tacrolimus, (**30**)) involve suppressing neuroinflammatory responses and promoting neuronal survival in SCI models. It reduces microglial activation, inhibits NF-κB signalling, decreases pro-inflammatory cytokine production (IL-1β, IL-6, TNF-α), and it enhances neuronal viability, altogether leading to a more permissive regenerative microenvironment [90]. Many compounds display dual antioxidant and anti-inflammatory effect through this convergent mechanism of enhancing Nrf2 activity coupled with downregulating NF-κB. This is particularly common in polyphenols (e.g., artepillin C, (**55**)) where redox and inflammatory regulatory targets modulation was coupled with neurite outgrowth in NGF-deprived PC12 Cells [91]. Multiple other mechanisms may also be involved. For example, the mushroom product, ganoderic acid A (**18**) showed anti-inflammatory and enhancement of regeneration markers such as BDNF to promotes remyelination in multiple sclerosis but the effects in vivo were associate with activation of farnesoid-X-receptor (FXR) [92]. While suppression of unregulated inflammation is critical in neuroregeneration, initial inflammation to clear injury is the critical aspect of the healing process following injury. In addition to promoting the survival and outgrowth of cultured Schwann cells in vitro, arecoline (**38**) has been shown to enhance peripheral (sciatic) nerve recovery by stimulating local inflammatory conditions [93].

Overall, the Nrf2/NF-κB upstream antioxidant and anti-inflammatory effects are closely associated with shifts in microglial and macrophage functional states, often described in the literature as a transition from pro-inflammatory M1-like phenotypes toward anti-inflammatory, pro-repair M2-like phenotypes. This immunological reprogramming is a key intermediate step linking biochemical permissiveness with structural neuroregeneration, as it reduces cytotoxic inflammatory signalling while enhancing phagocytic clearance, tissue remodelling, and trophic support. Interestingly, several NPs directly modulate this immunological switch. Salidroside (**47**) promotes neuronal repair and functional recovery through multiple mechanisms, including inhibition of astrocyte polarisation, reduction of glial scar formation, and simultaneous enhancement of neural stem cell proliferation and neuronal differentiation in SCI models [94]. Astragaloside IV (**58**) has been shown to promote microglia/macrophage M2 polarisation following cerebral ischemia/reperfusion injury, while also enhancing neurogenesis and angiogenesis, thereby coupling immune modulation with structural repair processes [95]. Mechanistically, this effect is linked to activation of the PPARγ (peroxisome proliferator-activated receptor-γ) signalling pathway, which promotes microglial/macrophage phenotypic switching toward the anti-inflammatory M2 state and simultaneously promotes neurogenic and angiogenic gene programmes that support tissue regeneration and functional recovery [95]. The mushroom metabolite, cordycepin (**17**), as a well-established anti-inflammatory compound can suppress microglial activation, through which it promotes proliferation of primary hippocampal neurons in vitro [96]. It also improved neuronal synaptic plasticity and senescence by promoting microglial polarisation toward the M2 phenotype which was shown to be CREB-induced NGF upregulation leading to symphony communication between the microglia and neuron in an Alzheimer’s disease (AD) model [97]. In a neonatal rat model of hypoxia-ischemia, butyrate (**68**) (representing SCFAs of the gut microbiota product), showed neurogenic effect via inhibition of inflammation, as evidenced by transitioning microglia to the anti-inflammatory phenotype (M2); while also increasing BDNF-TrkB signalling [98]. Immune-mediated effects such as neutrophil chemotaxis have also been suggested as the mechanism for the gut bacteria-derived metabolite indole-3-propionic acid (**72**) in promotion of nerve regeneration and repair in vivo [99]. An interesting insight on immunomodulation’s role in neurogenesis also comes from research on cyclosporin A (**32**) where it was shown to have direct effects on adult neural precursor cells both in vitro and in vivo. It does not influence the proliferation profile of neural precursor cells nor did it alter their differentiation but it promotes cell survival, resulting in increased numbers and larger colonies of neural precursor cells [100]. 

### 4.3. Metabolic Competence: Fuelling Regenerative Growth

Following establishment of a permissive microenvironment, neuroregeneration enters an energetically demanding phase characterised by cellular growth, biosynthesis, and structural remodelling. Neurite outgrowth, neuroblast migration, and cell maturation require substantial ATP to support cytoskeletal assembly, membrane expansion, protein synthesis, and intracellular transport. As a result, successful regeneration depends not only on removal of inhibitory signals but also on sufficient metabolic capacity to sustain repair [101]. To meet these demands, neurons and neural progenitor cells undergo metabolic adaptation involving enhanced mitochondrial oxidative phosphorylation, regulated glucose utilisation, and dynamic control of metabolic flux to support growth, differentiation, and tissue reconstruction [102]. Injury-associated mitochondrial dysfunction can limit regenerative capacity through impaired energy production, increased ROS generation, and disruption of intracellular signalling cascades. Maintenance and restoration of mitochondrial integrity therefore represent key determinants of regenerative competence. In this context, AMPK functions as a cellular energy sensor, sirtuin 1 (SIRT1) regulates stress adaptation and metabolic reprogramming, and PGC-1α promotes mitochondrial biogenesis [103]. Altogether, these pathways coordinate mitochondrial expansion and bioenergetic optimisation required for sustained structural growth. Through these mechanisms, regenerating cells expand mitochondrial capacity and optimise bioenergetic performance during prolonged regenerative activity. Not surprisingly, many NPs frequently converge on these same regulatory systems.

In cellular models (N2a/APP cells) and synaptic impairment assays, gastrodin (**51**) improves mitochondrial biogenesis and function through activation of the AMPK/SIRT1/PGC-1α axis, alongside its antioxidant and anti-inflammatory effects [104]. Similarly, in injured mature neurons in vitro and sciatic nerve injury models in vivo, harmine (**37**) promotes axon regeneration by enhancing mitochondrial energy supply via PGC-1α-dependent mitochondrial biogenesis [105]. Ginsenoside Rb1 (**56**) improves energy metabolism following SCI by restoring cellular energy balance and enhancing mitochondrial function, thereby supporting neuronal survival and functional recovery [106]. Mechanistically, it engages AMPK signalling and improves mitochondrial biogenesis and efficiency, consistent with broader evidence that ginsenosides enhance axonal regeneration by improving mitochondrial transport and reversing energy deficits [107]. Arecoline (**38**) exerts neuroprotective effects against ROS-induced oxidative injury in SH-SY5Y cells by reducing oxidative stress, limiting apoptosis, and stabilising mitochondrial function [108]. Similarly, luteolin (**42**) improves mitochondrial function, reduces oxidative stress, and decreases apoptosis in HT22 hippocampal neurons following isoflurane exposure [109]. Resveratrol (**46**) also supports structural and functional recovery following SCI through oxidative stress buffering and mitochondrial protection [68].

In addition to energy production, regenerating cells must maintain efficient intracellular recycling systems to preserve organelle integrity and sustain metabolic flexibility. Autophagy plays a central role in this process by degrading damaged proteins, membranes, and organelles, thereby recycling the substrates required for biosynthesis and repair. Selective autophagy processes, particularly mitophagy, are essential for removing dysfunctional mitochondria and preserving mitochondrial efficiency during regenerative stress [110]. Autophagy therefore represents a critical interface between cellular quality control, metabolic adaptation, and structural regeneration. Among NPs, urolithin A (**75**) is the best example, as a key regulator of autophagy-dependent neuroregeneration. It promotes peripheral nerve regeneration by enhancing transcription factor EB (TFEB)-mediated mitophagy and suppressing the NLRP3 inflammasome, thereby reducing inflammatory secondary injury and improving axonal regeneration following peripheral nerve damage [81]. Mechanistically, urolithin A restores mitochondrial quality control through TFEB activation, enhances mitophagic clearance of damaged mitochondria, and integrates lysosomal-autophagy regulation with suppression of inflammatory signalling (NLRP3/IL-1β axis), all leading to improvement in energy homeostasis and supporting axonal regrowth. Resveratrol (**46**) also activates autophagy-related pathways, as evidenced by increased expression of key markers (LC3-II and Beclin-1), leading to improved cellular clearance and stress adaptation following SCI [69]. Similarly, epothilones (e.g., (**33**)) facilitate peripheral nerve regeneration by promoting Schwann cell autophagy and migration in sciatic nerve injury models. In hippocampal neurons, berberine (**34**) promotes regenerative outcomes through autophagy-related signalling (involving LC3B, ATG7, and ATG16L1), alongside modulation of apoptotic regulators such as Bcl-2 and Bcl-w [111]. Autophagy is also implicated in the neuroregenerative effects of paeoniflorin (**52**) [112], further supporting its broad role across multiple NPs classes. The well-established flavonoid EGCG (**43**) histone deacetylase modulator has been shown to activate SIRT1 leading to autophagic response that attribute to neuroprotection in primary neurons [113]. Specifically, EGCG increases the acetylation of histone proteins via class III histone deacetylase. Altogether, these findings highlight that metabolic fuel availability, mitochondrial biogenesis, and autophagy-mediated quality control form an integrated energetic framework that is essential for sustaining neuronal growth, structural repair, and effective neuroregeneration.

### 4.4. Structural Growth Activation: Promoting Neurogenesis, Neurite Formation and Axonal Extension

#### 4.4.1. Intrinsic Growth Activation and Coordination

Following restoration of a permissive microenvironment and sufficient metabolic support, successful neuroregeneration requires activation of intrinsic neuronal growth programmes. This stage represents the central regenerative phase, during which neurons and neural progenitors actively generate new cellular structures, extend neurites and axons, and mature towards functional integration. Unlike upstream anti-inflammatory or antioxidant mechanisms, these processes directly promote tissue reconstruction and are therefore particularly relevant when evaluating the neuroregenerative potential of natural products.

Many NPs exert their primary regenerative actions through modulation of neurotrophic signalling pathways. Increased expression of neurotrophic factors, particularly BDNF, is among the most frequently reported mechanisms. Elevated BDNF levels promote neuronal survival, differentiation, neurite outgrowth and synaptic development through activation of its high-affinity receptor, TrkB. Most NPs act indirectly by enhancing endogenous neurotrophin production, whereas a select few exhibit direct neurotrophic or TrkB-associated activity, thereby stimulating downstream regenerative cascades independently of endogenous neurotrophin availability. Activation of Trk receptors initiates several growth-promoting signalling pathways, including PI3K/Akt, MAPK/ERK, cAMP/PKA/CREB, mTOR and GSK3β-regulated networks. As already stated in Section 1 and Section 2, these pathways coordinate transcriptional programmes required for neuronal differentiation, axonal elongation, dendritic arborisation and synaptic maturation while simultaneously supporting neuronal survival.

The growth and survival pathways most consistently reported as targets of NPs include PI3K/Akt as a central survival signalling axis, mTOR as a master regulator of protein synthesis and anabolic growth, MAPK/ERK as a regulator of differentiation and neurite extension, CREB as an activity-dependent transcription factor, cAMP/PKA as an intracellular signalling hub, and GSK3β as a key regulator of neuronal growth and structural plasticity. The number of NPs reported to influence these pathways in neuroregenerative models is exceptionally large, suggesting convergence upon a relatively restricted set of intracellular growth programmes despite considerable structural diversity among compounds.

Among mushroom NPs, *Hericium erinaceus* (Lion’s Mane mushroom) has emerged as one of the most extensively studied sources of neurotrophic agents. Extracts and bioactive constituents (**11**–**16**) stimulate neurite outgrowth, proliferation and differentiation of neural stem and progenitor cells, enhance neuronal survival under oxidative and excitotoxic conditions, and increase expression of neurotrophic factors including NGF and BDNF [114]. The aromatic compounds hericenones (hericenones C-E, (**11**–**13**)) promote neuritogenesis in PC12 cells through activation of PI3K/Akt and MAPK/ERK signalling pathways while potentiating endogenous NGF activity [115]. Similarly, the cyathane diterpenoids (e.g., erinacine C (**15**) and S (**16**)) activate transcriptional programmes involved in neurotrophic support and glial-neuronal communication while engaging downstream PI3K/Akt and MAPK/ERK signalling associated with neuronal maintenance and regeneration [116]. Other compounds such as ergothioneine (**21**) have been shown to enhance the release of BDNF induced by other agents [117] while ergothioneine (**21**) was also shown to induce neuronal stem cell differentiation via TrkB signalling [118].

Other mushroom-derived compounds capable of promoting neuronal growth include psychedelic secondary metabolites (e.g., psilocin (**19**) or psilocybin (**20**)), which induce structural and functional neural plasticity through enhanced dendritic spine formation, synaptogenesis and neuronal connectivity. These effects are mediated through 5-HT2A receptor activation, triggering BDNF-dependent signalling and downstream engagement of mTOR, PI3K/Akt and MAPK/ERK pathways [119]. Microbial-derived compounds also similarly converge on growth-promoting pathways. Rapamycin (**29**), originally isolated from *Streptomyces* species, remains one of the most informative examples linking mTOR signalling to regenerative responses. Rapamycin (**29**) modulates neurogenesis and synaptic reorganisation following cortical impact injury through regulation of mTOR-dependent growth pathways [120]. The importance of this pathway is further supported by evidence that TBI stimulates neural stem cell proliferation through activation of mTOR signalling [121]. Another microbial-derived macrolide, FK506 (tacrolimus, (**30**)), promotes peripheral nerve regeneration through coordinated regulation of mTOR, PI3K/Akt, MAPK/ERK, STAT3 and c-Jun signalling networks [122]. This list is however larger and include lovostatin (**31**) that upregulated neurotrophic signalling, including BDNF/TrkB and NGF/TrkA leading activating pro-survival pathways such as PI3K/Akt and MAPK/ERK as shown in TBI model in rats [123,124].

Representing marine NPs, sargaquinoic acid (**24**) isolated from brown alga, *Sargassum macrocarpum*, has been shown to induce neurite outgrowth in cultured PC12D cells via kinase A (not PKC)-mediated pathway. The study further showed the involvement of TrkA-MAP kinases and adenylate cyclase-PKA pathways leading to this effect [125]. Several plant-derived triterpenoids similarly converge on these intracellular growth programmes. Ginsenoside Rb1 (**56**) promotes axonal regeneration and motor recovery following stroke through activation of the cAMP/PKA/CREB signalling cascade, resulting in transcriptional induction of genes associated with neuronal survival, axonal growth and synaptic plasticity, including neurotrophic mediators such as BDNF [126]. Ginsenoside Rg1 (**57**) activates the BDNF/TrkB-ERK signalling axis following SCI, promoting neurogenesis, neuronal survival and plasticity through downstream MAPK/ERK-dependent transcriptional regulation [127]. The ginsenoside metabolite compound K (**76**) similarly modulates PI3K/Akt and MAPK/ERK pathways while enhancing neurotrophic support and neuronal integration of newly generated cells [128]. The triterpene mushroom product, ergosterol (**22**), promotes neurite outgrowth in neuronal cell culture subjected to amyloid-β (Aβ), by increasing the phosphorylation of the ERKs activity of cAMP response element (CRE), and GAP-43 [129]. Withanolide A (**60**) with steroidal lactone structure stimulates neurite regeneration and synaptic reconstruction through activation of pro-growth signalling pathways including MAPK/ERK and PI3K/Akt while also facilitating cytoskeletal remodelling required for neurite extension [130]. Andrographolide (**59**) is a diterpene example of compounds that promote adult hippocampal neurogenesis through activation of PI3K/Akt and MAPK/ERK pathways and also enhances neurotrophic signalling [131,132]. Another diterpene example is ginkgolide B (**61**) which features a unique structure with six five-membered rings, three trilactones and 4-hydroxy groups. It enhances proliferation and differentiation of neural stem cells following cerebral ischemia/reperfusion injury through modulation of PI3K/Akt and MAPK/ERK pathways alongside increased neurotrophic support [133]. The most striking example of terpenoids as neuroregenerative agent comes from the action of limonene (**64**) as a monoterpene, which despite its simple structure, has been demonstrated to display neuroregenerative effects [79]. The study showed that, beyond anti-inflammatory activity, it increases the level of NGF, GAP-43 and the phosphorylated ERK.

The polyphenolic constituents of plants also frequently engage neurotrophic growth pathways. Luteolin (**42**) promotes neurogenesis and neuronal survival in hippocampal models through increased expression of BDNF, NT-3, TrkB and phosphorylated CREB [66]. Additional studies demonstrated increased BDNF and phosphorylated ERK1/2, supporting activation of the BDNF/ERK pathway during regenerative responses [134]. In human neural stem cells, luteolin (**42**) further promoted neuronal differentiation while increasing BDNF, TrkB and phosphorylated-CREB expression [135]. Salidroside (**47**) similarly promotes neuroregeneration through enhanced neurotrophic support. Following ischemic brain injury, salidroside increased the expression of neurotrophic factors including BDNF, NGF and NT-3 while improving endogenous neural regeneration [136]. Consistent findings have been reported in Schwann cell cultures and sciatic nerve injury models, where salidroside increased NGF and BDNF expression along with enhanced proliferation and regenerative activity [137,138]. Salvianolic acid B (**50**) directly stimulates adult neural stem/progenitor cells and enhances neurogenesis both in vitro and in vivo. These effects were associated with activation of BDNF/TrkB and NGF/TrkA signalling together with downstream PI3K/Akt and MAPK/ERK pathways that support survival, proliferation and differentiation of neural progenitor populations [139]. In addition to ERK1/2, artepillin C (**55**) modulates p38 MAPK pathways which regulate cytoskeletal dynamics, gene transcription, and growth cone remodelling, thereby facilitating neurite initiation and elongation as demonstrated in neuronal-like cells in vitro [140]. As a functional outcome, many phenolics, including thymol (**65**), have been robustly reported to increase the proliferation of cultured neuronal cells such as in primary hippocampal cultures [141]. In addition to promoting the proliferation, differentiation, and survival of neural stem cells in vitro, tanshinone IIA (**67**) was also observed to promote hippocampal neurogenesis in ApoE^−/−^ mice possibly through the cAMP/PKA/CREB/BDNF signalling pathway [142]. Improvement of neural tissue recovery in SCI model by gastrodin (**51**) was also associated with increased secretion of BDNF [143]. In peripheral nerves, these effect on BDNF (as well as neurofilament-200 and myelin basic protein) release was shown to be associated with suppression of miR-497 [144]. The study showed that restoring BDNF levels could reverse the negative impacts of miR-497, highlighting the emerging role of the miR-497/BDNF axis in nerve regeneration. Hence, miR-497 overexpression is a therapeutic target as it suppresses the proliferation and migration of Schwann cells while also exacerbating oxidative stress [144]. While promoting endothelial cell proliferation and tube-like formation via upregulation of VEGF expression in vitro, astragaloside IV (**58**) supports recovery in ischemic stroke by promoting angiogenesis and PI3K/Akt/mTOR signalling pathway [145].

Modulation of GSK3β is frequently linked to activation of Wnt/β-catenin signalling which serves as a major regulator of neural stem cell proliferation and neuronal differentiation. Salvianolic acid A (**49**) promotes endogenous neurogenesis following ischemic stroke through increased Wnt3a expression, suppression of GSK3β activity, and stabilisation of β-catenin, thereby activating Wnt-dependent transcriptional programmes associated with neuronal growth and lineage commitment [146]. Similar regulation has been reported for oleanolic acid (**63**), which inhibits GSK3β and promotes accumulation of β-catenin, resulting in activation of Wnt/β-catenin-dependent neurogenic signalling pathways [147]. Andrographolide (**59**) also induced neural differentiation of rat adipose tissue-derived stromal cells by activating the Wnt/β-Catenin signalling pathway [148]. In addition to growth-associated kinase pathways, NPs frequently influence injury-response transcriptional programmes that determine regenerative competence. Key targets include STAT3, a central regulator of regeneration-associated gene expression; c-Jun, which coordinates injury-response transcriptional networks; ATF3 (activating transcription factor 3), a stress-responsive transcription factor strongly associated with axonal regeneration; and p300 acetyltransferase, an epigenetic regulator that promotes transcriptional activation of growth-supportive genes. FK506 (**30**) has been shown to regulate injury-associated transcription factors including STAT3 and c-Jun while simultaneously reducing fibroblast survival and promoting extracellular matrix remodelling, thereby improving the regenerative microenvironment following peripheral nerve injury [90,149]. Similarly, resveratrol (**46**) activates p300 acetyltransferase, leading to enhanced VEGF (vascular endothelial growth factor) signalling, neuronal survival, angiogenesis and axonal regeneration in rat sciatic nerve crush injury models [150]. Altogether, these observations suggest that NPs influence not only canonical growth pathways but also the transcriptional and epigenetic programmes that determine the capacity of injured neurons to initiate and sustain regenerative growth. In human neural progenitor cells derived from pluripotent stem cells, harmine (**37**) induces a cell proliferative effect associated with selective inhibition of DYRK1A (dual-specificity tyrosine phosphorylation-regulated kinase 1A) [151]. This effect underpins the evidence that upregulated DYRK1A kinase on impairment of neurogenesis and its involvement in NDs such as in AD makes it a key target.

Finally, a number of compounds influence downstream growth-associated proteins that function as molecular correlates of regenerative activity. Quercetin (**41**) promotes neurite growth through increased intracellular cAMP levels and upregulation of GAP-43, a protein strongly associated with axonal growth and growth cone activity [152]. Similar responses have been reported for EGCG (**43**), which increases GAP-43 expression while simultaneously enhancing anti-apoptotic Bcl-2 signalling and reducing Bax expression, thereby promoting neuronal survival and axonal regeneration [153]. Comparable increases in growth-associated proteins and regenerative markers have also been reported for ursolic acid (**62**) during peripheral nerve regeneration [154]. Other NPs such as butyrate (**68**) which inhibits HDACs can regulate cell proliferation and differentiation by inducing bHLH as shown in several experimental models [155]. This was also evidenced in vivo where it increased the activity of the TrkB receptor and the phosphorylation of the transcription factor, CREB in the ipsilateral hemisphere [98]. Receptor mediated-cell proliferation effects are also noted for NPs. For example, (−)-stepholidine (**40**), an effective dopamine D1 receptor agonist and D2 receptor antagonist, has been shown to promotes proliferation and neuronal differentiation of rat embryonic striatal precursor cells in vitro [156].

#### 4.4.2. Overcoming Extrinsic Growth Inhibition

In contrast to the large number of NPs reported to activate neurotrophic signalling (PI3K/Akt, MAPK/ERK, mTOR, CREB, etc.) and related pro-growth pathways, relatively few NPs have been shown to directly modulate the canonical extracellular inhibitory mechanisms that restrict regeneration within the injured CNs system. These inhibitory systems include myelin-associated inhibitors, glial scar-associated signalling pathways, growth-suppressive transcriptional programmes, and intracellular mediators of growth cone collapse such as the RhoA/ROCK axis. Nevertheless, several notable examples demonstrate that NPs can partially overcome these regenerative barriers.

Among the best-characterised examples is the diterpenoid quinone tanshinone IIA (**67**), which promotes axonal regeneration following focal cerebral ischemia through inhibition of the Nogo-A/NgR1/RhoA/ROCKII/MLC signalling pathway. Tanshinone IIA suppresses the expression of Nogo-A and its receptor NgR1, resulting in downstream inhibition of RhoA/ROCK signalling and reduced myosin light-chain activation. Such effects alleviate cytoskeletal contraction and growth cone collapse, thereby facilitating axonal extension and structural plasticity within inhibitory environments [157]. Similarly, the microbial macrolide epothilone D (**33**) promotes axonal sprouting following CNS injury through stabilisation of microtubules and modulation of growth cone-associated cytoskeletal machinery. Experimental studies have implicated targets associated with RhoA/ROCK signalling, cytoskeletal assembly, axonal transport, and growth cone dynamics, resulting in improved regenerative growth following transection and stroke injury models [158,159].

At a broader tissue level, salidroside (**47**) has been reported to improve neuronal repair and functional recovery following SCI through mechanisms that include reduction of glial scar formation together with enhanced neural stem cell proliferation and neuronal differentiation [91,160]. Although the precise molecular mechanisms are not defined, these findings suggest modulation of structural barriers that normally restrict regenerative growth. Another recurrent target is the Notch signalling pathway, which can contribute to glial activation, maintenance of inhibitory cellular states and suppression of regenerative differentiation programmes following injury. In an SCI model, resveratrol (**46**) promoted tissue repair and functional recovery through inhibition of Notch signalling, thereby relieving inhibitory constraints on neuronal regeneration [161]. Similar modulation of Notch-dependent pathways has been reported for salidroside (**47**), whose regenerative effects following ischemic brain injury involve suppression of Notch signalling alongside increased neurotrophic factor expression [136]. Likewise, paeoniflorin (**52**) suppresses maladaptive Notch signalling in experimental tethered spinal cord injury [112], while curcumin (**7**) promotes adult neural stem cell proliferation and neuronal lineage commitment through regulation of Notch-dependent cell fate programmes [162]. Similar effects have also been described for carvacrol (**66**), which mitigates fluoride-induced impairment of neurogenesis through modulation of Notch1 signalling [163].

In addition to extracellular inhibitory pathways, several NPs target intracellular suppressors of regenerative growth. Luteolin (**42**) protects hippocampal neurons through regulation of the miR-214/PTEN/Akt axis, preventing miR-214 downregulation, suppressing PTEN activity and restoring Akt phosphorylation [109]. Similarly, astragaloside IV (**58**) promotes neuronal axon regeneration through inhibition of PTEN, a major endogenous negative regulator of PI3K/Akt signalling. By relieving PTEN-mediated suppression of Akt activation, astragaloside IV enhances downstream pro-survival and growth-associated signalling pathways involved in cytoskeletal remodelling and axonal extension [164].

Several NPs also influence cellular stress-adaptation mechanisms that indirectly improve regenerative competence. Resveratrol (**46**) enhances neuronal survival and functional recovery following SCI through activation of autophagy-related pathways, including increased expression of LC3-II and Beclin-1, suggesting enhanced cellular clearance and adaptation to injury-associated stress [69]. Although autophagy is not traditionally considered a canonical extrinsic inhibitory pathway, improved proteostatic and metabolic homeostasis may enhance the capacity of neurons to respond to inhibitory environments. All these observations suggest that a subset of NPs appear to be capable of modulating major inhibitory systems including Nogo-A/NgR1/RhoA/ROCK signalling, Notch-dependent suppression, PTEN-mediated growth restriction, glial scar formation and injury-associated stress responses. Such mechanisms may complement neurotrophic and metabolic programmes by reducing the extracellular and intracellular constraints that normally prevent successful regenerative growth within injured nervous tissue.

Limited research is also available on the proliferative potential of NPs on Schwann cells. Of these, the best example is the alkaloid, fascaplysin (**23**), derived from the marine sponge *Fascaplysinopsis berguist*, with fused benzoyl-linked β-carboline skeleton [165]. Other structurally related compounds of marine origin (e.g., 9-methylfascaplysin) have been shown to promote neurite outgrowth at nanomolar concentrations via the inhibition of ROCK2 as evidenced in PC12 cells [166]. This study also used molecular docking analysis which demonstrated a high binding affinity with ROCK2.

### 4.5. Functional Integration and Adaptive Plasticity

Following structural regeneration, newly generated neurons and regenerating axons must be incorporated into existing neural circuits to enable functional recovery. This stage represents the transition from anatomical repair to functional network restoration, in which newly formed connections must be stabilised and recruited into coherent activity patterns. However, when compared with upstream neuroprotective and neurite-outgrowth mechanisms, successful functional integration and circuit-level rewiring remain comparatively less well characterised in NPs research and represent a major unresolved challenge in neuroregeneration. 

At present, most evidence for NPs in this late phase is indirect, emerging from studies reporting improved functional outcomes alongside modulation of neurotransmitter systems and injury recovery markers rather than direct demonstration of circuit reconstruction. In TBI models, galantamine (**36**) exerts neuroprotective and recovery-associated effects through modulation of acetylcholinesterase (AChE) and nicotinic acetylcholine receptors (nAChRs) (Zhao et al., 2018) [167]. Similarly, paeoniflorin (**52**) promotes neuronal repair following cerebral ischemia-reperfusion injury via nicotinic acetylcholine receptor-mediated signalling (α7 and α4β2 nAChRs), leading to reduced inflammatory burden and improved recovery outcomes [75]. Carvacrol (**66**) has also been associated with improved neurological outcomes following TBI, consistent with broader recovery-linked neuroprotective effects [78]. While NPs can support recovery-associated functional improvements, as evidenced by these findings, direct evidence for precise synaptic reintegration and long-range circuit reconstitution still remains limited, highlighting this stage as a critical gap in current neuroregeneration research.

### 4.6. Other Group of NPs with Demonstrated Neuroregenerative Effect Through Diverse and Emerging Mechanisms

Readers should note that several NPs have been reported to enhance neuroregeneration despite their mechanisms of action remaining incompletely defined. These include several dietary factors and endogenous metabolites such as polyamines. Spermidine (**71**) is a representative example, with substantial experimental evidence supporting its neuroregenerative potential across multiple model systems. In PC12 cells, spermidine promoted neurite outgrowth, increasing the proportion of cells extending neurites at least twice the diameter of the cell body, both in the presence and absence of NGF [168]. Recently, spermidine has also been shown to improve mitochondrial bioenergetics in both young and aged human induced pluripotent stem cell-derived neurons by increasing ATP production and mitochondrial membrane potential, reducing mitochondrial ROS, and enhancing mitochondrial respiration [169]. In a mouse model of optic nerve injury, dietary spermidine reduced retinal ganglion cell death, attenuated retinal degeneration, suppressed activation of the ASK1 (apoptosis signal-regulating kinase 1)-p38 MAPK signalling pathway, decreased chemokine production and microglial recruitment, and enhanced optic nerve regeneration [170]. Additional evidence implicates endogenous polyamine synthesis in regenerative responses, as increased arginase I activity promotes putrescine production, which is subsequently converted to spermidine. Spermidine was shown to be necessary for overcoming myelin-associated inhibitors of regeneration and was sufficient to promote optic nerve regeneration in vivo, identifying polyamine metabolism as a critical component of the conditioning lesion response [171]. Thus, experimental evidence indicates that spermidine promotes neuroregeneration through multiple complementary mechanisms, including enhancement of neurite outgrowth, preservation of mitochondrial function, modulation of inflammatory signalling, and regulation of endogenous polyamine metabolism, although the primary molecular targets responsible for these effects remain to be fully elucidated.

Readers should also note that there are several emerging pharmacological targets through which several NPs act to promote neuroregeneration. For example, the membrane-bound Eph/ephrin (erythropoietin-producing human hepatocellular receptors and their receptor-interacting proteins) signalling system serves as a major molecular brake in the adult CNS following trauma, and its upregulation collapses axonal growth cones and promotes glial scarring to physically block regeneration. Some NPs are now emerging to bypass this barrier, by acting as a direct small-molecule antagonist that physically binds and blocks the EphA4 receptor [172]. By silencing this target’s downstream cytoskeletal collapse signals, such compounds seem to transform themselves from general neuroprotection agents into active neuroregeneration compounds at the circuit wiring stage. Semaphorin 3A (Sema3A) is another emerging target for NPs bypassing the glial scar. Post-trauma, secreted Sema3A binds axonal neuropilin-1 (NRP1)/PlexinA1 receptors leading to signalling for collapse of growth cones. Some fungal metabolites are now emerging to act as direct Sema3A antagonists to promote axonal regeneration [173]. Beyond the plant-derived polyphenols acting upstream via NF-κB inhibition to downregulate astrocyte CSPG secretion, marine-derived fucosylated chondroitin sulphates act as structural decoys to mask or counteract the inhibitory GAG matrix, successfully promoting neurite outgrowth [174]. As discussed in Section 4.2, many NPs acting globally as redox regulators have direct relevance in neuroregeneration by promoting a permissive environment and exerting neuroprotective effects. In this direction, NPs heavily modulate HIF-1α (hypoxia-inducible factor 1-α) to promote hypoxic adaptation, while concurrently upregulating GCLC and GCLM (glutamate-cysteine ligase catalytic and modifier subunits) to reset intracellular redox balance. Such effects are not elaborated further as they are mostly regarded as neuroprotective mechanisms in CNS pathologies.

## 5. Understanding the Complex Neuroregenerative Pathway of Natural Products: The Targets–Mechanisms–Networks (T-M-N) Analysis Model

Despite the large and continually expanding number of NPs reported to promote neuroregenerative responses across experimental models detailed in Section 4, this body of studies have not produced a corresponding breakthrough in novel drug discovery. As outlined in Section 2, this translational gap cannot be attributed to a single competing limitation. Improvements in bioavailability and tissue exposure have not, by themselves, overcome the intrinsic pharmacological constraints of many NPs, while adequate exposure does not necessarily ensure sufficient target engagement, potency, appropriate temporal or spatial signalling, compatibility with the stage-dependent injury environment, or convergence of the multiple processes required for regeneration. The central question therefore shifts from whether individual NPs can produce regenerative responses to what recurring biological functions they engage, how these functions interact, and whether their collective activity converges on the network states required for regeneration. This provides the rationale for the following Target–Mechanism–Network (T-M-N) analysis, in which the diverse NPs identified across experimental models are examined not as isolated compounds, but as pharmacological probes of the molecular and cellular systems governing neuroregeneration.

**Figure 12:**
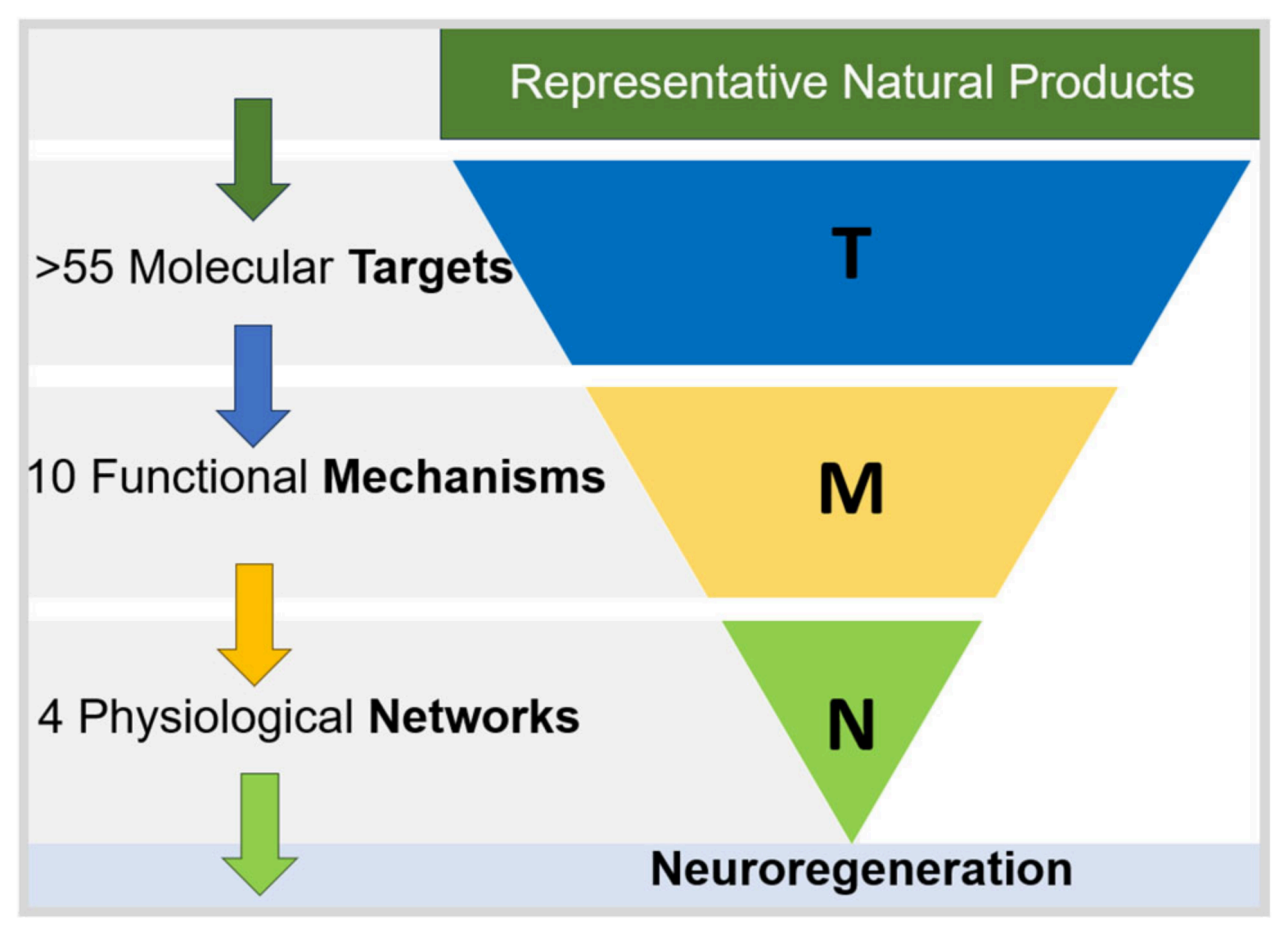
Progressive convergence from chemical diversity to neuroregenerative system states within the T-M-N analysis model. Close to 80 representative NPs were examined in the literature to map the observed 55 molecular targets, 10 mechanistic modules (M1–M10), and 4 network-level states (N1–N4). The funnel illustrates the progressive emergence of functional convergence across biological scales, showing how chemically diverse natural products ultimately act through a limited set of conserved neuroregenerative control systems.

Systematic synthesis of the neuroregenerative NPs literature across both in vitro and in vivo studies reveals a convergent multi-scale regulatory architecture which is extensively discussed in Section 4. Across diverse NP classes, reported molecular and cellular effects can be consistently mapped onto a hierarchical organisation spanning molecular targets, functional mechanisms, and higher-order network behaviours. At the lowest level, more than 55 recurrent molecular targets (T) can be identified from the literature and organised into ten intermediate functional mechanisms (M) representing shared regulatory processes (Table 1). These mechanisms further integrate into four network-level control regimes (N1–N4) governing energy availability, regenerative signalling competence, redox-immune homeostasis, and structural plasticity (Table 2). These regulatory layers constitute a T-M-N architecture that provides a structured representation of the distributed biology underlying neuroregeneration, as summarised in the accompanying funnel diagram (Figure 12; >55 targets → 10 mechanisms → network control regimes).

The T-M-N analysis approach is not intended as a new mechanistic approach but as a synthesis model that compresses a highly fragmented literature into a tractable organisational hierarchy. By reducing numerous reported molecular interactions into progressively higher levels of functional abstraction, the analysis enables comparison across structurally distinct NPs and highlights recurring patterns of biological convergence. Instead of interpreting neuroregenerative activity through individual pathways or isolated targets, the T-M-N-architecture captures how diverse molecular perturbations are integrated into common functional outputs and ultimately into system-level regenerative states. This hierarchical representation transforms a large and heterogeneous body of evidence into a coherent model of regenerative control and provides a basis for identifying common organisational principles underlying NP-mediated neuroregeneration.

Integration of the ten mechanistic modules into four higher-order systems (N1–N4) further reveals the functional architecture of neuroregeneration. It defines interdependent regulatory domains whose coordinated activity determines regenerative outcome. The N1 (Energy Network) establishes the metabolic and mitochondrial capacity required to support regenerative demands. The N2 (Growth Network) governs the activation and execution of neuronal survival, neurotrophic, and axonal growth programmes. The N3 (Redox-Immune Network) regulates the permissive or inhibitory regenerative environment through the integration of oxidative stress responses, inflammatory signalling, glial activation states, and stress-adaptive regulatory mechanisms. The N4 (Plasticity and Structural Network) controls the capacity of neural tissues to undergo adaptive remodelling through coordinated regulation of extracellular matrix dynamics, vascular support, injury-responsive transcriptional programmes, and structural plasticity. These networks and/or systems overall represent emergent functional states instead of discrete pathways, providing a systems-level framework through which diverse molecular perturbations can be interpreted in terms of their contribution to regenerative competence.

Within this architecture, successful neuroregeneration is not viewed as the consequence of activating individual molecular targets or signalling pathways, but as the coordinated establishment of an energetically competent, growth-permissive, stress-resilient, and structurally adaptable cellular state. The significance of the T-M-N-model therefore lies not only in organising mechanistic knowledge but also in revealing a conserved systems/network-level structure that appears repeatedly across chemically diverse neuroregenerative NPs. This observation provides the conceptual foundation for subsequent deconstruction analyses (Section 6), where recurring structural motifs can be evaluated according to their contribution to specific mechanistic modules, network control regimes, and regenerative system states, thereby enabling the rational identification of chromophores suitable for pseudo-natural product design. For instance, neurotrophin-associated scaffolds such as hericenone and erinacine A exhibit a structurally distinct capacity to engage neurotrophic signalling networks (Section 4), with reported activity converging on PI3K/Akt-associated survival pathways and the neurotrophin receptor-linked signalling axis. This dominant signalling route maps onto the M2/M7 mechanistic modules, which integrate into the N2 Growth System governing neuronal survival, axonal extension, and regenerative signalling competence. At the same time, downstream transcriptional activation of these compounds via CREB-associated signalling represents a secondary but functionally reinforcing regulatory layer, linking neurotrophic input to metabolic and survival-supportive processes associated with M1 and M3 modules, thereby leading to both N1 (energetic readiness) and N2 (growth-permissive signalling capacity). This dual-level engagement reflects a characteristic feature of neurotrophic NPs, where a primary growth-axis activation is coupled to broader metabolic and stress-adaptive reinforcement within the regenerative state space. On the regulatory control side, rapamycin exemplifies higher-order modulation of neuroregenerative capacity through mTOR-dependent growth control and cellular stress-response integration (Section 4). Instead of directly activating neurotrophic signalling cascades, rapamycin functions primarily as a growth-gating and resource-allocation regulator to influence the balance between anabolic protein synthesis, autophagy, and metabolic conservation. Through inhibition of mTORC1 signalling, it constrains the activation state of M2/M7-associated regenerative mechanisms, thereby indirectly shaping the extent and temporal dynamics of engagement within the N2 Growth System. In this regard, N2 is not defined solely by pathway activation, but as an emergent state resulting from the coupling of growth-promoting signals with metabolism and permissiveness governed by upstream regulatory targets such as mTOR. Similarly, the NP examples described above can be systematically mapped onto the molecular targets outlined in Section 1, which collectively define the three foundational pillars of overcoming neuroregeneration barriers. Within the T-M-N analysis, these mappings can reveal that structurally diverse compounds converge on distinct but interrelated target nodes governing neurotrophic activation, growth control, and stress-metabolic regulation, thereby providing mechanistic entry points into the M- and N-layers of the regenerative architecture. These NPs thus occupy defined positions within a shared T-M-N topology, reinforcing the concept of neuroregeneration as a coordinated systems-level process.

As a note of caution, target attribution for NPs should be interpreted in light of the well-recognised potential for assay interference and promiscuous activity, particularly among polyphenolic compounds such as curcumin, quercetin, EGCG, resveratrol and luteolin. Reported modulation of a target or signalling pathway was therefore considered within the experimental context of the original study and does not necessarily imply direct or selective molecular engagement. Where orthogonal target-validation evidence was unavailable, these observations were treated as experimentally reported target/pathway associations instead of definitive single-target mechanisms. This distinction is important for interpreting the T-M-N framework, which is intended to capture recurring functional mechanisms and network convergence across the literature rather than to imply equivalent target specificity or pharmacological certainty for every NP.

## 6. Network-Guided Deconstruction of Natural Products for Bioactive Motif Discovery

As discussed in Section 3, PNP strategies are being explored in drug discovery as a means of expanding chemical space beyond the structures and pharmacological limitations of native NPs. By recombining or modifying structural elements derived from NPs, these approaches can generate molecules with altered target engagement, improved pharmacological properties, or biological activities not observed in the parent compounds. Their application within the CNS, however, remains comparatively limited, and their potential in neuroregeneration has received particularly little systematic investigation. Nevertheless, several early examples provide proof-of-principle for applying NP-inspired design to neurobiological targets (Section 3). A preliminary study using in vitro system showed that two compounds (HJ-01 and HJ-02) were rationally designed from illudalic acid, a fungal NPs that inhibits protein tyrosine phosphatase sigma (PTPσ). Interestingly, the designed compounds did not simply reproduce the parent mechanism. Illudalic acid inhibited PTPσ catalytic activity but did not rescue axon outgrowth, whereas HJ-01/HJ-02 did not inhibit PTPσ catalytic activity at biologically relevant concentrations but disrupted the PTPσ–Trk interaction, enhanced Trk signalling and restored sympathetic axon outgrowth across inhibitory CSPGs [175]. Another preliminary example is based on paecilomycine A, a fungal NP with neurotrophic activity, but its structural complexity motivated the development of simpler NP-inspired scaffolds. The resulting compounds were evaluated for neurotrophic and neurogenic properties. In particular, one paecilomycine A-inspired compound showed neurotrophic activity in neuronal cells and ex vivo neurogenic activity in neonatal mouse hippocampal neurosphere cultures. Subsequent work explored their neuroactive properties in the context of stroke therapeutics [176]. Beyond the CNS, the recent development of NP-derived induced-proximity molecules such as the iDeg series (Section 3) further illustrates how NP fragments can be converted into compounds with pharmacological mechanisms distinct from those of their parent molecules. All these studies establish the feasibility of PNP design as drug-discovery strategy, even though direct evidence for such compounds producing long-distance axonal regeneration, synaptic reintegration, or regeneration-specific functional recovery remains very limited. This gap provides the rationale for exploring whether the mechanistic information derived from the NPs neuroregeneration literature can be systematically translated into a network-guided PNP design strategy.

The central design of the PNP strategy involves the fusion of biosynthetically unrelated NP fragments to create novel chemotypes with unique biological activities [64]. In their review of the evolution of NPs-inspired drug discovery strategies, Cremosnik et al. [177] highlighted the transition from biology-oriented synthesis to PNPs as an effective means of accessing novel and biologically relevant chemical space. As discussed in Section 3, numerous studies have demonstrated the value of this approach in drug discovery, with proof-of-concept already established across several therapeutic areas. In light of these findings, along with the central outcome of the present cross-mechanistic analysis of NPs, the PNP strategy represents a promising methodology for the discovery of next-generation neuroregenerative compounds. By enabling the rational exploration of biologically relevant yet previously inaccessible chemical space, this approach may facilitate the identification of molecules with enhanced neuroregenerative potential and novel mechanisms of action.

The main outcome of the present analysis is that neuroregenerative NPs do not function as isolated pharmacological entities but to repeatedly converge upon a finite and recurrent set of biological processes encompassing inflammation and oxidative stress resolution, metabolic reprogramming, mitochondrial support, neurogenesis, axonal growth, synaptic repair, and cellular resilience. Across structurally diverse compound classes, remarkably consistent functional convergence is observed over a wide range of experimental injury and disease models despite substantial chemical heterogeneity. Moreover, this convergence is not accompanied by single-target specificity as most neuroregenerative NPs exhibit distributed polypharmacology. They simultaneously engage multiple interconnected pathways including Nrf2-NF-κB-mediated redox regulation, AMPK/SIRT1/PGC-1α-dependent metabolic adaptation, and pro-regenerative signalling networks governing cytoskeletal remodelling, axonal transport, neuronal survival, and plasticity. While such pleiotropic activity likely underpins their broad therapeutic potential, it also presents a translational challenge, as biological effects are frequently modest, pathology-dependent, and difficult to attribute to discrete molecular mechanisms.

When viewed from a systems/network-level perspective, however, the apparent complexity of the NPs analysis in neuroregenerative research becomes a valuable source of design information. The hierarchical integration of biological activity data from individual molecular targets to functional mechanisms and ultimately to network-level processes provides a rational framework for identifying bioactive motifs suitable for PNP design. Organising neuroregenerative biology into T-M-N layers reveals recurring structural motifs embedded within chemically diverse NPs that consistently modulate multiple targets of the same regenerative network. This network-based approach of NP classification draws principles from phenotypic drug discovery [178] and biology-oriented synthesis [179] in that it puts its focus more on the biology outcomes than single molecular targets. The desired biological phenotype, such as neuroregeneration using validated NP scaffolds as starting points for chemical design, can be targeted by identifying NPs that produce similar phenotypic outcomes and extracting shared structural motifs. In this way, it may be possible to generate novel PNP enriched for the desired biological activity.

Recent advances in multi-omics technologies have further transformed NP discovery from traditional bioactivity-guided isolation towards data-driven approaches that integrate genomics, transcriptomics, proteomics, metabolomics, and phenomics. These methodologies enable the identification of bioactive metabolites through the analysis of complex biological systems, linking molecular profiles to phenotypic outcomes and uncovering previously inaccessible regions of biologically relevant chemical space [180]. By facilitating the systematic discovery of compounds associated with desired biological states, multi-omics approaches may provide a powerful framework for identifying privileged NP fragments, scaffolds, and bioactive structures that could inform the rational design of next-generation PNPs.

The bioactive motifs identified through convergent biological network analysis may be repurposed as modular building blocks for PNP construction, retaining the polypharmacological properties of the parent natural products while improving control over potency, selectivity, physicochemical properties, and synthetic accessibility. The network-guided bioactive motif selection may also extend beyond single-target optimisation to the rational design of compounds capable of modulating the multifactorial biology underlying neurodegeneration. This strategy is strengthened by phenotypic screening approaches that capture integrated cellular responses relevant to neuroregeneration. High-content imaging platforms, including multiplexed neuronal-glial profiling and cell painting assays, can enable simultaneous quantification of diverse cellular features such as neurite morphology, synaptic integrity, mitochondrial function, and glial activation states. The cell painting assay, in particular, provides a highly multiplexed morphological fingerprint of cellular state and has been widely used for mechanism-agnostic profiling of bioactive small molecules [181]. Altogether, these multidimensional phenotypic signatures form a functional bridge between network-informed NPs deconstruction and chemical space exploration, enabling prioritisation of bioactive fragments combinations that reproduce integrated regenerative states far beyond isolated pathway modulation. This provides a practical route for translating systems-level NPs insights into the rational design of next-generation neuroregenerative PNPs.

## 7. Conclusions

Across five decades of experimental neuroregeneration research, NPs have consistently demonstrated robust neuroprotective activity and reproducible partial regenerative effects in diverse in vitro and in vivo models. However, the collective evidence indicates that their translational limitations cannot be fully attributed to PK or delivery constraints alone, as even when CNS exposure is achieved through direct administration or advanced delivery strategies, sustained and functionally meaningful structural regeneration remains limited. This suggests that a more fundamental constraint lies in intrinsic biophysical properties governing signalling potency, temporal coordination, and multi-scale integration within the injured central nervous system. Neuroregeneration is not driven by single linear pathways but by coordinated, sequential biological programmes encompassing inflammatory resolution, metabolic activation, axonal extension, and circuit integration. While NPs exhibit widespread activity across these domains modulating redox and inflammatory states, metabolic adaptation, and cytoskeletal and plasticity-associated signalling, these effects are typically distributed across multiple molecular targets and rarely integrated within a single coherent pharmacological entity capable of sustaining the full regenerative sequence. This distributed activity becomes more formally resolved when mapped onto a T-M-N architecture derived from systematic synthesis of the neuroregenerative literature. Across diverse NP classes, approximately 55 recurrent molecular targets can be grouped into 10 functional mechanisms, which in turn converge into four higher-order network control regimes governing energy availability, regenerative signalling competence, redox-immune balance, and structural plasticity. This hierarchical organisation reveals extensive convergence at the level of network control despite substantial heterogeneity at the level of individual targets and pathways. In this context, NPs do not act as unified multi-stage regenerative agents, but as distributed modulators that partially engage different components of a shared neuroregenerative network architecture without fully spanning it. Mapping NP activity onto this T-M-N model resolves the apparent convergence paradox: structurally diverse compounds repeatedly access the same regenerative networks, yet no single scaffold appears to comprehensively coordinate all network states required for complete functional recovery. This reframes NPs not as end-stage therapeutics, but as a pre-validated fragment space encoding evolutionarily selected pharmacophores that selectively engage discrete components of neuroregenerative network control. This review provides a direct rationale for a PNP strategy, in which structurally and biosynthetically unrelated NP-derived fragments are recombined into engineered scaffolds designed to enhance signalling coherence across the T-M-N hierarchy. By moving from distributed NP activity to integrated network engagement, this approach aims to align pharmacological output with the multi-stage requirements of central nervous system repair, enabling the design of compounds with improved potency, functional continuity, and regenerative completeness.

## Figures and Tables

**Table 1:** Major molecular targets and functional mechanisms (observed) in NPs neuroregenerative research *.

Code	Target (T)	Target Function	Functional Mechanism (M) *
T1	AMPK	Cellular energy sensing	M1—Energy and Metabolism Cellular energy sensing, mitochondrial biogenesis, metabolic adaptation, and stress response.
T2	SIRT1	Stress adaptation/metabolic regulation	
T3	PGC-1α	Mitochondrial biogenesis	
T4	PPARγ	Metabolic/inflammatory switching	
T5	PI3K/Akt	Survival signalling	M2—Growth and Survival Signalling Intracellular signalling pathways promoting cell survival, growth, differentiation, and plasticity.
T6	mTOR	Protein synthesis/anabolic growth	
T7	MAPK/ERK	Differentiation and growth	
T8	CREB	Activity-dependent transcription	
T9	cAMP/PKA	Intracellular signalling hub	
T10	GSK3β	Growth and plasticity regulator	
T11	BDNF	Synaptic plasticity	M3—Neurotrophic System Major neurotrophin-mediated support for neuronal survival, axonal growth, and synaptic plasticity.
T12	TrkB	BDNF receptor signalling	
T13	NGF	Neuronal survival	
T14	TrkA	NGF receptor signalling	
T15	NT-3	Axonal guidance and myelination	
T16	TrkC	NT-3 receptor signalling	
T17	STAT3	Regenerative transcription	M4—Injury-Response Transcription Transcriptional and epigenetic activation in response to injury or stress to promote regenerative programmes.
T18	c-Jun	Injury-response gene activation	
T19	ATF3	Stress-induced regeneration	
T20	p300 acetyltransferase	Epigenetic transcriptional activation	
T21	HIF-1α	Hypoxic adaptation	M5—Redox, Hypoxic adaptation and Antioxidant Defence Antioxidant response, hypoxic adaptation, angiogenesis, and cytoprotective redox regulation.
T22	VEGF	Angiogenesis/vascular remodelling	
T23	Nrf2	Antioxidant response	
T24	HO-1/NQO1	Cytoprotective redox enzymes	
T25	GCLC/GCLM	Glutathione synthesis	
T26	NF-κB	Inflammatory signalling	M6—Neuroimmune and Inflammation regulation Regulation of inflammation, microglial and astrocyte response, cytokine signalling, and immune resolution.
T27	NLRP3 inflammasome	Innate immune activation	
T28	Cytokines (IL-1β, IL-6, TNF-α)	Pro-inflammatory signalling	
T29	JAK/STAT	Cytokine signalling	
T30	Microglial activation	Innate immune modulation	
T31	Astrocyte reactivity	Glial response	
T32	Complement system	Synaptic pruning	
T33	Immune resolution	Transition to repair	
T34	RhoA/ROCK/MLC	Cytoskeletal inhibition axis	M7—Axonal Growth Machinery Cytoskeletal dynamics, growth cone modulation, axonal transport, and GAP-43-mediated axonal growth programmes.
T35	Growth cone dynamics	Axon extension	
T36	Cytoskeletal assembly	Neurite formation	
T37	Axonal transport	Intracellular trafficking	
T38	GAP-43/cAMP-GAP-43 axis	Axonal growth programme	
T39	Nogo-A/NgR1	Myelin inhibition	M8—Extrinsic Inhibition Extracellular and myelin-associated inhibitors, glial scar formation, and repulsive guidance signals.
T40	CSPGs	ECM inhibition	
T41	Semaphorins	Axon repulsion	
T42	Eph/ephrin	Connectivity guidance	
T43	Glial scar formation	Physical regeneration barrier	
T44	HDACs	Chromatin regulation	M9—Epigenetic Regulation Chromatin, DNA methylation, and miRNA regulation controlling gene expression and plasticity.
T45	DNA methylation	Gene silencing control	
T46	miRNA regulation	Post-transcriptional regulation	
T47	miR-214/miR-497	Plasticity and apoptosis regulation	
T48	ECM remodelling	Structural reorganisation	M10—Structural and Plasticity Environment Extracellular matrix, BBB integrity, cellular reprogramming, autophagy, stem cell signalling, and permissive plasticity.
T49	ECM stiffness	Mechanical signalling	
T50	BBB integrity	Vascular barrier regulation	
T51	Cell fate conversion	Cellular reprogramming	
T52	Wnt/β-catenin	Stem/plasticity signalling	
T53	Notch signalling	Differentiation control	
T54	TFEB/LC3-II/Beclin-1	Autophagy-lysosomal clearance	
T55	nAChRs (α7/α4β2)	Cholinergic plasticity signalling	

* Major mechanistic targets (T-codes) observed in the various neuroregeneration research on NPs are mapped onto higher-order functional mechanisms (M-codes). Regenerative phenotypes such as neurite outgrowth, axonal regeneration, synaptic plasticity, remyelination, angiogenesis, autophagy, and mitophagy are excluded as primary targets and are considered emergent system-level effects arising from integrated multi-target regulation. At least 55 targets can be selected from the neuroregenerative literature for the present analysis which are grouped under 10 mechanisms which represent a broad functional layer encompassing related signalling, regulatory, and cellular processes. Substantial cross-talk exists between modules, particularly between redox and neuroimmune regulation, gene expression and growth signalling, and immune and axonal growth systems. However, these interactions are not explicitly encoded at this level to preserve structural clarity. The mechanisms are defined to reflect dominant functional groupings to provide a simplified systems-level organisation that supports downstream integration and interaction analysis. AMPK, adenosine monophosphate-activated protein kinase; ATF3, activating transcription factor 3; Astrocyte reactivity, astrocyte activation and hypertrophic response; BBB integrity, blood–brain barrier; BDNF, brain-derived neurotrophic factor; cAMP/PKA, cyclic adenosine monophosphate/protein kinase A pathway; c-Jun, transcription factor c-Jun protein; CREB, cAMP response element-binding protein; CSPGs, chondroitin sulphate proteoglycans; ECM, extracellular matrix; Eph/ephrin, erythropoietin-producing human hepatocellular receptors and corresponding ligands; GAP-43/cAMP-GAP-43, growth-associated protein 43/cyclic AMP regulatory signalling; GCLC/GCLM, glutamate–cysteine ligase catalytic and modifier subunits; GSK3β, glycogen synthase kinase 3 beta; HDACs, histone deacetylases; HIF-1α, hypoxia-inducible factor 1-alpha; HO-1/NQO1, haeme oxygenase-1/NAD(P)H quinone dehydrogenase 1 antioxidant; IL, interleukin; JAK/STAT, Janus kinase/signal transducer and activator of transcription; MAPK/ERK, mitogen-activated protein kinase/extracellular signal-regulated kinase; miRNA, microRNA; mTOR, mammalian target of rapamycin; nAChRs, nicotinic acetylcholine receptors; NF-κB, nuclear factor kappa light chain enhancer of activated B cells; NGF, nerve growth factor; NLRP3 inflammasome, NOD-like receptor pyrin domain-containing 3 inflammatory multiprotein complex; Nogo-A/NgR1, neurite outgrowth inhibitor A/Nogo receptor 1 inhibitory system; Nrf2, nuclear factor erythroid 2-related factor 2; NT-3, neurotrophin-3; p300 acetyltransferase, histone acetyltransferase p300 protein; PGC-1α, peroxisome proliferator-activated receptor gamma coactivator 1-alpha; PI3K/Akt, phosphoinositide 3-kinase/protein kinase B pathway; PPARγ, peroxisome proliferator-activated receptor gamma; RhoA/ROCK/MLC, ras homolog family member A/Rho-associated protein kinase/myosin light chain inhibitory pathway; Semaphorins, chemorepulsive guidance glycoproteins; SIRT1, sirtuin 1 NAD-dependent deacetylase; STAT3, signal transducer and activator of transcription 3; TFEB/LC3-II/Beclin-1, transcription factor EB/microtubule-associated protein 1 light chain 3 beta-II/Beclin-1 autophagic pathway; TrkA, tropomyosin receptor kinase A; TrkB, tropomyosin receptor kinase B; TrkC, tropomyosin receptor kinase C; TNF, tumour necrosis factor alpha; VEGF, vascular endothelial growth factor; Wnt/β-catenin, wingless-related integration site/beta-catenin transcriptional pathway.

**Table 2:** Network-Level integration architecture of neuroregenerative NPs (N1–N4) *.

Code	Integrated M-Modules	Description
N1—Energy Network	M1	Governs cellular ATP production, mitochondrial function, and metabolic capacity that determines baseline energetic readiness for regenerative processes.
N2—Growth Network	M2, M3, M7	Integrates survival signalling pathways, neurotrophic systems, and axonal growth machinery controlling neuronal viability and structural regeneration.
N3—Redox-Immune Network	M5, M6, M8, M9	Integrates oxidative stress regulation, inflammatory signalling, glial responses, extrinsic growth inhibition, and epigenetic stress-immune coupling that determine regenerative permissiveness or suppression.
N4—Plasticity and Structural Network	M4, M10	Integrates injury-responsive gene expression, extracellular matrix remodelling, vascular integrity, and cellular plasticity constraints governing tissue-level adaptability and structural reorganisation.

* The table presents a hierarchical model for natural products-modulated neuroregeneration, integrating functional modules (M-codes) into four higher-order networks (N1–N4).

## Data Availability

No new data were created or analysed in this study. Data sharing is not applicable to this article.
